# The metabolomic signature of weight loss and remission in the Diabetes Remission Clinical Trial (DiRECT)

**DOI:** 10.1007/s00125-023-06019-x

**Published:** 2023-10-25

**Authors:** Laura J. Corbin, David A. Hughes, Caroline J. Bull, Emma E. Vincent, Madeleine L. Smith, Alex McConnachie, Claudia-Martina Messow, Paul Welsh, Roy Taylor, Michael E. J. Lean, Naveed Sattar, Nicholas J. Timpson

**Affiliations:** 1 MRC Integrative Epidemiology Unit at University of Bristol, Bristol, UK; 2 Population Health Sciences, Bristol Medical School, University of Bristol, Bristol, UK; 3 School of Translational Health Sciences, Dorothy Hodgkin Building, University of Bristol, Bristol, UK; 4 Robertson Centre for Biostatistics, Institute of Health and Wellbeing, University of Glasgow, Glasgow, UK; 5 School of Cardiovascular and Metabolic Health, University of Glasgow, Glasgow, UK; 6 Newcastle Magnetic Resonance Centre, Translational and Clinical Research Institute, Newcastle University, Newcastle upon Tyne, UK; 7 Human Nutrition, School of Medicine, Dentistry and Nursing, College of Medical, Veterinary & Life Sciences, University of Glasgow, Glasgow, UK

**Keywords:** Diabetes remission, DiRECT, Metabolomics, Randomised controlled trial, Type 2 diabetes, Weight loss

## Abstract

**Aims/hypothesis:**

High-throughput metabolomics technologies in a variety of study designs have demonstrated a consistent metabolomic signature of overweight and type 2 diabetes. However, the extent to which these metabolomic patterns can be reversed with weight loss and diabetes remission has been weakly investigated. We aimed to characterise the metabolomic consequences of a weight-loss intervention in individuals with type 2 diabetes.

**Methods:**

We analysed 574 fasted serum samples collected within an existing RCT (the Diabetes Remission Clinical Trial [DiRECT]) (*N*=298). In the trial, participating primary care practices were randomly assigned (1:1) to provide either a weight management programme (intervention) or best-practice care by guidelines (control) treatment to individuals with type 2 diabetes. Here, metabolomics analysis was performed on samples collected at baseline and 12 months using both untargeted MS and targeted ^1^H-NMR spectroscopy. Multivariable regression models were fitted to evaluate the effect of the intervention on metabolite levels.

**Results:**

Decreases in branched-chain amino acids, sugars and LDL triglycerides, and increases in sphingolipids, plasmalogens and metabolites related to fatty acid metabolism were associated with the intervention (Holm-corrected *p*<0.05). In individuals who lost more than 9 kg between baseline and 12 months, those who achieved diabetes remission saw greater reductions in glucose, fructose and mannose, compared with those who did not achieve remission.

**Conclusions/interpretation:**

We have characterised the metabolomic effects of an integrated weight management programme previously shown to deliver weight loss and diabetes remission. A large proportion of the metabolome appears to be modifiable. Patterns of change were largely and strikingly opposite to perturbances previously documented with the development of type 2 diabetes.

**Data availability:**

The data used for analysis are available on a research data repository (https://researchdata.gla.ac.uk/) with access given to researchers subject to appropriate data sharing agreements. Metabolite data preparation, data pre-processing, statistical analyses and figure generation were performed in R Studio v.1.0.143 using R v.4.0.2. The R code for this study has been made publicly available on GitHub at: https://github.com/lauracorbin/metabolomics_of_direct.

**Graphical Abstract:**

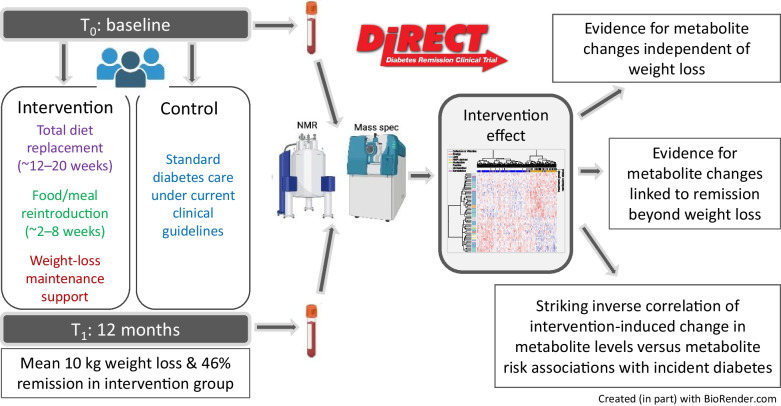

**Supplementary Information:**

The online version contains supplementary material available at 10.1007/s00125-023-06019-x.


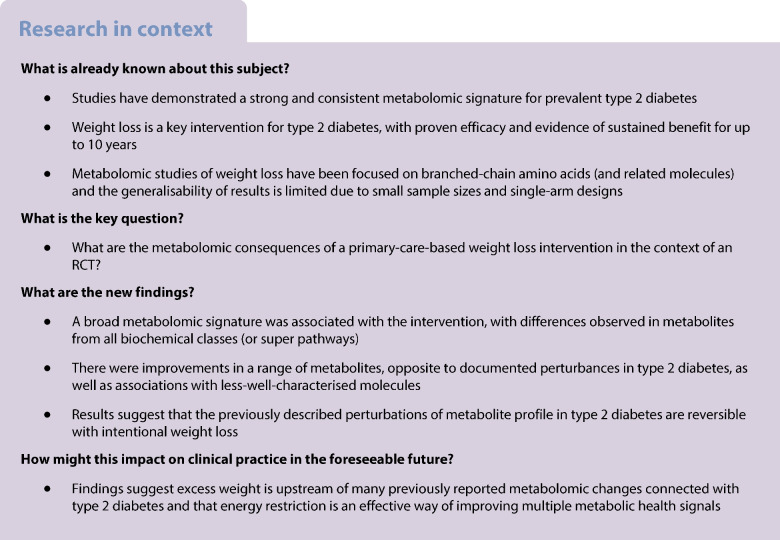


## Introduction

For conditions like type 2 diabetes where there is a clear relationship between risk factors, intermediate metabolic phenotypes and disease, attention has turned to metabolomics as a potentially useful tool for elucidating the biological mechanisms underpinning disease pathology [1, 2]. Studies to date have demonstrated a strong and consistent metabolomic signature of prevalent type 2 diabetes [3] and incident disease [4, 5]. Unsurprisingly, given the strong overlap in the metabolomic signature of type 2 diabetes and its precursors (overweight/obesity and insulin resistance) [6, 7], many of the metabolomic perturbations observed in individuals diagnosed with disease also appear to have a role in disease development [8, 9]. However, it remains to be determined whether the changes observed reflect a systemic ‘downstream’ response to high glucose per se or else ‘upstream’ excess adiposity [10, 11].

To help elucidate the role of metabolites in type 2 diabetes, studies that evaluate the metabolomic response to disease remission following interventions are critical. Weight loss is a key intervention for individuals with type 2 diabetes, with proven efficacy and evidence of sustainability of the metabolic benefits for up to 10 years [12, 13]. While there have been metabolomics studies of weight loss, the reliability and robustness of results published to date have been questioned (e.g. due to small sample sizes and single-arm designs) [14]. There remains a need to characterise the molecular underpinnings of existing interventions targeting diabetes remission through weight loss. Accordingly, we took samples from a seminal RCT involving an intensive weight management programme, the Diabetes Remission Clinical Trial (DiRECT), with the aim of characterising the metabolomic footprint of this intervention.

## Methods

### Study design and participants

DiRECT was a 2 year open-label, cluster RCT conducted at 49 primary care practices in Scotland and the Tyneside region of England between 25 July 2014 and 5 August 2016 (isrctn.org registration no. ISRCTN03267836). The trial was carried out to assess whether effective weight management, delivered in a primary care setting, could produce sustained remission of type 2 diabetes. The protocol has been published elsewhere [15], as have the baseline characteristics of the groups [16]. In brief, general practices were randomly assigned (1:1) to provide either a weight management programme (intervention) or best-practice care by guidelines (control), with stratification for study site (Tyneside or Scotland) and practice list size (>5700 or ≤5700). Individuals aged 20–65 years who had been diagnosed with type 2 diabetes within the past 6 years, had a BMI of 27–45 kg/m^2^ and were not receiving insulin were recruited. The intervention (Counterweight-Plus, https://www.counterweight.org/) comprised withdrawal of glucose-lowering and antihypertensive drugs, total diet replacement (3452–3569 kJ [825–853 kcal]/day formula diet for 3–5 months), stepped food reintroduction (2–8 weeks) and structured support for long-term weight-loss maintenance. Ethics approval was granted by West 3 Ethics Committee in January 2014, with approvals by the National Health Service (NHS) health board areas in Scotland and clinical commissioning groups in Tyneside. All participants provided written informed consent.

The trial was conducted over a period of 2 years with principal data collection points scheduled at baseline, 12 months and 2 years. Blood samples were collected and a range of clinically relevant outcomes measured, including liver function tests, cholesterol and triglycerides (TGs) [15]. In this study, we analysed samples from the baseline and 12 month time points using both an untargeted MS approach (Metabolon, Durham, NC, USA) and ^1^H-NMR spectroscopy (Nightingale Health, Finland). For all other data used in our analyses, we used the same version of the trial database as used for the main trial analysis at 12 months, as reported by Lean et al [17]. These data comprised an intention-to-treat population of 149 participants per group (total *N*=298).

### Sample collection and metabolite data acquisition

Participants were asked to fast overnight before the blood draw. Sample handling procedures are described in electronic supplementary material (ESM) Methods. In total, 574 serum samples collected from 302 unique individuals during the trial were sent for metabolomic analysis. All analysts were blinded to intervention/control status. Samples were sent first to Metabolon. The dataset returned (‘MS data’) included 1276 metabolite features comprising 959 compounds of known identity (named biochemicals with the majority matched to purified standards) and 317 compounds of unknown structural identity (unnamed biochemicals, indicated by a superscript ‘a’ in the main-text tables). Remaining sample material was then sent to the MRC Integrative Epidemiology Unit Metabolomics Facility (University of Bristol) for ^1^H-NMR analyses (after one further freeze–thaw). The dataset returned (‘NMR data’) included 148 primary measures quantified in absolute concentrations as well as 79 additional ‘derived measures’ such as ratios and percentages. Further details of the metabolite data acquisition can be found in ESM Methods.

### Metabolite data preparation

Data quality checks were carried out locally using a pre-release version of the R package metaboprep [18] with samples and features excluded from subsequent statistical analysis based on a pre-defined set of quality control (QC) metrics. Full details of the procedures are given in ESM Methods and data summaries produced are included within the associated GitHub repository (https://github.com/lauracorbin/metabolomics_of_direct). Data were restricted to include only those individuals present in the trial database (*N*=298) and for whom both a baseline (T_0_) and 12 month follow-up (T_1_) sample were present in the filtered metabolite data. Two processed datasets were derived: (1) RNT dataset, for which metabolite data were transformed (across individuals within timepoint) using a rank-based inverse normal transformation (where tied ranks were split by assigning a random order); and (2) PA dataset, for which metabolite data were transformed to a presence/absence phenotype such that missing values were replaced with 0 and non-missing values (i.e. those with an abundance measure) were replaced with 1.

### Statistical analysis

**Fig. 1:**
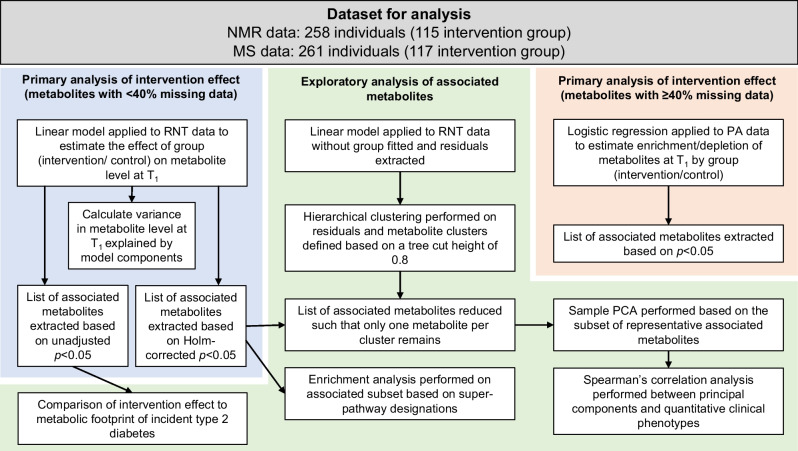
Statistical analysis overview. PA, data transformed to a presence/absence phenotype with missing values replaced with 0 and non-missing values with 1; RNT, data transformed using a rank-based inverse normal transformation; T_1,_ 12 month time point (post intervention)

An overview is shown in Fig. 1. We analysed all available data according to group allocation with the control group as the reference and effect estimates therefore representing the difference in the intervention group relative to the control group.

#### Linear regression model

In our evaluation of the effect of the intervention on metabolite levels, outcomes (metabolite levels at T_1_) were compared between groups with linear regression models applied to the RNT dataset. Where metabolite_T0_ or metabolite_T1_ was missing (unquantified) for an individual, that individual was excluded from the analysis for that specific metabolite yielding varied sample sizes across metabolites. Models were adjusted for study centre and practice list size along with the baseline measurement of the outcome (metabolite at T_0_), age and sex, all fitted as fixed effects:

$${\mathrm{metabolite}}_{\mathrm{T}1} \sim \mathrm{ study}.\mathrm{centre }+\mathrm{ list}.\mathrm{size }+\mathrm{ age }+\mathrm{ sex }+ {\mathrm{metabolite}}_{\mathrm{T}0} +\mathrm{ allocation}$$

where metabolite_T1_ is metabolite level at 12 months, ‘study centre’ is a binary variable (Tyneside or Scotland), ‘list size’ is a binary variable indicating practice list size (>5700 or ≤5700), age is participant age at baseline (years), sex is a binary variable (male/female), metabolite_T0_ is metabolite level at baseline and allocation is a binary exposure variable indicating the individual’s treatment group (control or intervention). Model β represents the expected difference in metabolite level at T_1_ expressed as normalised SD units per unit difference in the dependent variable after adjusting for metabolite level at T_0_ (in the case of allocation this is the mean difference between groups in metabolite at T_1_). Results from this analysis were considered to be the primary result for all metabolite features with <40% missing (unquantified) data at the 12 month timepoint. This missingness threshold was selected based on the increase in the SE of the treatment group effect estimated from the linear model when the number of observations fell below this level (see ESM Fig. 1). The Holm [19] method was used to adjust *p* values for multiple testing and an adjusted *p* value of <0.05 was considered as evidence for association. Extended methods relating to the linear regression model can be found in ESM Methods.

#### Pathway enrichment analyses

Hypergeometric-based enrichment analyses were conducted to evaluate the enrichment of classes in the subset of associated features derived from the linear model as compared with all features that were tested by the same model. Metabolite super-pathway designation provided by Metabolon was used for the enrichment analysis, with NMR-derived metabolites allocated to super pathways following the approach of Wahl et al [20].

#### Exploratory analysis of associated metabolites and clinical phenotypes

Where metabolites were found to be associated with the intervention, several follow-up analyses were performed (see ESM Methods for full details). To evaluate the extent to which the association between metabolite_T1_ and allocation could alternatively be explained by weight change, the primary model above was re-fitted with the addition of ‘weight change’ as a fixed effect. To begin to understand the potential relevance of metabolite levels to diabetes remission, over and above their role as proxies for weight loss, metabolite change was compared in individuals who did and did not achieve diabetes remission within quantiles of weight change. Finally, a principal component analysis (PCA)-based investigation was conducted into the relationship between the metabolites found to be associated with the intervention and the change in a subset of clinical phenotypes selected based on their relevance to the long-term health of individuals with type 2 diabetes.

#### Comparison of intervention and incident type 2 diabetes footprints

To assess the extent to which the changes we see here in response to the DiRECT intervention are equal and opposite to those observed prior to development of type 2 diabetes, we extracted summary relative risk (SRR) estimates from a recent meta-analysis of prospective cohort studies [5]. Metabolites were matched as far as possible against those with meta-analysis results (presented in Supplementary Table 7 of Morze et al [5]) using either Human Metabolome Database (HMDB) identifiers or biochemical names. Where the metabolite was measured by both platforms, the instance with the smallest *p* value was retained. Intervention effects (β extracted from the linear model) were plotted against log_*e*_ SRRs extracted from the meta-analysis. We focused the comparison on those metabolites where there was evidence of association with the intervention based on an unadjusted *p* value threshold of >0.05. Analysis with all matched metabolites is also made available.

#### Logistic regression model

A logistic model was applied to the PA datasets to compare the presence of each metabolite by allocation as follows:

$${\mathrm{metabolite}}_{\mathrm{T}1} \sim {\mathrm{metabolite}}_{\mathrm{T}0} +\mathrm{ allocation}$$

where metabolite_T1_ is metabolite presence at 12 months (1, present; 0, absent) and metabolite_T0_ is metabolite presence at baseline (1, present; 0, absent) and allocation is as defined above. In this exploratory model, no covariates were fitted to maximise the power of the test. Model βs for the exposure, ‘allocation’, represent the coefficient for intervention group, that is the log_*e*_ OR between intervention group and control group. Results from this analysis, in which we assume missing data are due to very low levels of the metabolite (below that which can be detected) or to complete absence of the metabolite, were considered the primary result for all features with ≥40% missing (unquantified) data. A *p* value of <0.05 was considered as evidence for association with no adjustment made for multiple testing.

## Results

### Study characteristics

At 12 months, mean body weight had fallen by 10 kg in the intervention group and by 1 kg in the control group (adjusted difference –8.8 [95% CI –10.3, –7.3] kg, *p*<0.0001) and almost half of the 149 participants in the trial arm (46%) had achieved type 2 diabetes remission (as defined in the trial protocol) as compared with six participants (4%) in the control arm [17]. Baseline characteristics were similar when comparing the intervention group with the control group in the subset of participants with metabolomics data (Table 1). Following in-house QC, the NMR data comprised 567 samples and 225 metabolic features (147 primary measures and 78 derived measures) and the MS data comprised 571 samples and 1254 metabolites (ESM Table 1). After merging with trial data there were 258 individuals in the NMR dataset (115 intervention and 143 control) and 261 individuals in the MS dataset (117 intervention and 144 control) available for statistical analysis.

**Table 1:** Baseline characteristics (*N*=261)

Characteristic	Intervention group (*n*=117)		Control group (*n*=144)	
	Mean	SD (%)	Mean	SD (%)
Sex, *n*				
Female	49	42	55	38
Male	68	58	89	62
Age, years	53.7	7.1	56.2	6.9
BMI, kg/m^2^	34.8	4.5	34.3	4.3
Weight, kg	100.3	16.8	99.0	16.0
Fasting glucose, mmol/l	9.3	3.2	8.8	2.6
Total cholesterol, mmol/l	4.3	1.1	4.3	1.1
HDL-cholesterol, mmol/l	1.1	0.3	1.2	0.3
TG, mmol/l	2.0	1.5	1.9	0.9

Summary statistics were calculated based on the MS sample (after QC) (*N*=261)

### Effect of intervention on metabolites: linear regression model

Results from the multivariable linear model formed the primary result for all 147 NMR metabolites and 78 NMR-derived measures with a minimum (median) sample size of 199 (258). Of the NMR metabolites tested at 12 months, 59 (26%) were altered by the intervention (Holm-corrected *p*<0.05) (including 27 derived measures) with 41 (69% of those altered) showing an increase in response to treatment (ESM Fig. 2, ESM Table 2). The strongest association was seen for glucose (β −0.71 [95% CI −0.92, −0.50], Holm-corrected *p*=3.77×10^−8^). Results from the linear model formed the primary result for 1064 (85%) of the MS metabolites, with a minimum (median) sample size of 93 (260). Of the metabolites tested, 127 (12%) were associated with the intervention (Holm-corrected *p*<0.05) with 72 (57% of those associated) showing an increase in response to treatment (ESM Fig. 2, ESM Table 3). The strongest association was seen for a metabolite identified as erythronate (β −0.82 [95% CI −0.99, −0.65], Holm-corrected *p*=2.84×10^−15^), although the identity of this metabolite has not yet been confirmed by Metabolon based on a standard. For most metabolites, there was little evidence for between-group (control/intervention) differences in levels at baseline; one out of 186 associated metabolites (4-hydroxychlorothalonil) had *p*<0.05/186 (Wilcoxon rank sum test) (for boxplots, see GitHub repository: https://github.com/lauracorbin/metabolomics_of_direct).

In the intervention group, we observed a decrease in phosphatidylethanolamines, branched-chain amino acids (BCAAs) and related metabolites (i.e. those allocated to the same super- and sub-pathways) and sugars, and in the relative abundance of TG to total lipids within specific lipid fractions (e.g. TG/total lipids ratio in small, medium and large LDL particles). In contrast, increases were seen in lipids including sphingolipids, plasmalogens and metabolites assigned to the ‘fatty acid metabolism (acyl choline)’ sub-pathway and for amino acids from the sub-pathways ‘glycine, serine and threonine metabolism’ and ‘urea cycle; arginine and proline metabolism’. There was also evidence (from NMR) for an increase in the intervention group of the proportion of cholesterol and cholesteryl esters relative to total lipids in a variety of lipid fractions and an increase in the ratio of polyunsaturated fatty acids to total fatty acids.

### Pathway enrichment analyses

Enrichment analyses gave evidence for enrichment in the associated metabolites for NMR-derived measures (2.4-fold, *p*=5.08×10^−6^) and for the carbohydrate super pathway (2.4-fold, *p*=0.011) (ESM Fig. 3). This suggests that metabolites allocated to these groups were overrepresented in the list of associated metabolites.

### Exploratory analysis of associated metabolites and clinical phenotypes

For the vast majority of metabolites examined, when weight change was added as a fixed effect to the primary linear model, the intervention effect on metabolite level at 12 months was attenuated, as demonstrated by a qualitative reduction in the variance explained by allocation (ESM Tables 2, 3). In a small number of cases (seven for NMR and one for MS), adjusting for weight change did not result in the attenuation of the intervention effect (e.g. ‘omega-3 fatty acids’ [*n*-3 fatty acids] [NMR] and sphingomyelin [d17:1/14:0, d16:1/15:0]^a^ [MS], where the superscript ‘a’ indicates that the compound has not been confirmed based on a standard). For some metabolites, there was evidence for metabolite change explaining additional variance in remission status beyond that explained by weight change (ESM Table 4). For example, a difference in mean metabolite change by remission status was seen within individuals in the first quantile of weight change (−31.6 kg to −9.0 kg) for 1,5-anhydroglucitol (*p*=8.52×10^–5^), MS-measured glucose (*p*=4.64×10^–5^) and other sugars (ESM Fig. 4). Although some of this difference may be attributable to residual variance in weight change (within quantiles), at least in the case of the aforementioned metabolites, the association of metabolite change with remission status remained after adjustment for weight change when fitted in a linear regression model (data not shown).

**Fig. 2:**
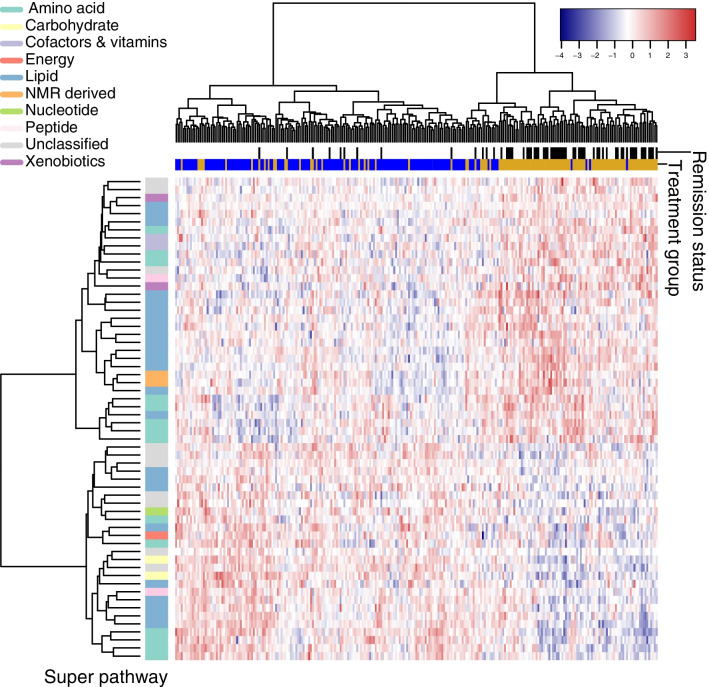
Clustering to show treatment group allocation and type 2 diabetes remission status. Heatmap shows metabolite levels at 12 months derived from covariate-adjusted RNT data for the 61 intervention-associated representative metabolites. Further description of plot generation can be found in ESM Methods. ‘Remission status’ indicates participant’s type 2 diabetes status at 12 months, such that white indicates no remission and black indicates remission. In the case of ‘treatment group’ allocation, blue indicates control and orange indicates intervention group

Prior to PCA, a hierarchical clustering approach allocated the 1289 metabolites with <40% missing data to 238 metabolite clusters (ESM Table 5). Using these clusters, the full list of 186 associated metabolites was reduced to a set of 61 approximately independent, representative features for use in the PCA, of which 51 had at least a putative identification (Table 2 and ESM Table 6). The PCA analysis (ESM Fig. 5 for resultant scree plot) exhibited separation of participants on principal component 1 (PC1; which explained 21% of the variance) according to both their allocation to intervention or control arms of the trial and their remission status at 12 months; this pattern is illustrated in Fig. 2. There was also evidence for a correlation between the metabolomic footprint of the intervention (as captured by the PCs) and clinical indicators of metabolic health (e.g. HbA_1c_), as well as several phenotypes relevant to non-alcoholic fatty liver disease (ESM Results, ESM Table 7 and ESM Figs 6A, B, 7).

**Table 2:** Metabolites associated with intervention from linear model (named/annotated representative features only)

Biochemical name	Super pathway	Sub-pathway	Source	β	Lower 95% CI	Upper 95% CI	Holm-corrected *p* value
Erythronate^a^	Carbohydrate	Aminosugar metabolism	Metabolon	−0.82	−0.99	−0.65	2.84×10^−15^
*N*-Lactoyl isoleucine	Amino acid	Leucine, isoleucine and valine metabolism	Metabolon	−0.87	−1.08	−0.66	5.96×10^−11^
1-(1-Enyl-palmitoyl)−2-oleoyl-GPC (P−16:0/18:1)^a^	Lipid	Plasmalogen	Metabolon	0.59	0.43	0.74	2.28×10^−9^
β-Alanine	Nucleotide	Pyrimidine metabolism, uracil containing	Metabolon	−0.68	−0.87	−0.49	9.86×10^−9^
Glucose	Carbohydrate	Glycolysis, gluconeogenesis and pyruvate metabolism	Metabolon	−0.75	−0.95	−0.54	2.13×10^−8^
1-Stearoyl−2-oleoyl-GPE (18:0/18:1)	Lipid	Phosphatidylethanolamine	Metabolon	−0.67	−0.86	−0.48	3.26×10^−8^
Glycosyl ceramide (d18:2/24:1, d18:1/24:2)^a^	Lipid	Hexosylceramides	Metabolon	0.64	0.46	0.82	5.16×10^−8^
Hydroxy-CMPF^a^	Lipid	Fatty acid, dicarboxylate	Metabolon	0.35	0.24	0.45	2.60×10^−7^
Sphingomyelin (d18:1/22:2, d18:2/22:1, d16:1/24:2)^a^	Lipid	Sphingomyelins	Metabolon	0.54	0.38	0.70	3.59×10^−7^
Isoleucine	Amino Acid	NA	Nightingale	−0.62	−0.82	−0.41	2.66×10^−6^
Betaine	Amino Acid	Glycine, serine and threonine metabolism	Metabolon	0.52	0.35	0.68	4.36×10^−6^
4-Ethylphenylsulfate	Xenobiotics	Benzoate metabolism	Metabolon	0.65	0.43	0.86	1.07×10^−5^
Alanine	Amino Acid	Alanine and aspartate metabolism	Metabolon	−0.63	−0.83	−0.42	1.14×10^−5^
Palmitoylcholine	Lipid	Fatty acid metabolism (acyl choline)	Metabolon	0.66	0.43	0.88	2.94×10^−5^
6-Bromotryptophan	Amino Acid	Tryptophan metabolism	Metabolon	0.57	0.37	0.77	6.49×10^−5^
3β,7α-Dihydroxy−5-cholestenoate	Lipid	Sterol	Metabolon	−0.41	−0.55	−0.26	1.12×10^−4^
4-Hydroxychlorothalonil	Xenobiotics	Chemical	Metabolon	0.36	0.23	0.50	2.84×10^−4^
Palmitoyl sphingomyelin (d18:1/16:0)	Lipid	Sphingomyelins	Metabolon	0.49	0.30	0.67	3.35×10^−4^
Cholesterol esters to total lipids ratio in medium VLDL	NMR ratio/percentage	NA	Nightingale	0.49	0.29	0.69	4.76×10^−4^
N-Acetylmethionine	Amino acid	Methionine, cysteine, SAM and taurine metabolism	Metabolon	0.51	0.31	0.71	7.35×10^−4^
Bilirubin (Z,Z)	Cofactors and vitamins	Haemoglobin and porphyrin metabolism	Metabolon	0.48	0.29	0.67	7.98×10^−4^
Arachidonoylcarnitine (C20:4)	Lipid	Fatty acid metabolism (acyl carnitine)	Metabolon	0.46	0.28	0.64	9.66×10^−4^
Tryptophan betaine	Amino acid	Tryptophan metabolism	Metabolon	0.49	0.29	0.68	1.70×10^−3^
3-Methyl−2-oxovalerate	Amino acid	Leucine, isoleucine and valine metabolism	Metabolon	−0.47	−0.66	−0.28	1.91×10^−3^
TGs in small HDL	Lipid	NA	Nightingale	−0.43	−0.62	−0.24	2.18×10^−3^
1-Arachidonoyl-GPC (20:4n6)^a^	Lipid	Lysophospholipid	Metabolon	0.46	0.27	0.64	2.22×10^−3^
Total cholesterol in very large HDL	Lipid	NA	Nightingale	0.39	0.22	0.56	2.46×10^−3^
α-Hydroxycaproate	Lipid	Fatty acid, monohydroxy	Metabolon	−0.62	−0.87	−0.37	2.47×10^−3^
Sphingomyelin (d17:1/14:0, d16:1/15:0)^a^	Lipid	Sphingomyelins	Metabolon	0.38	0.23	0.54	2.57×10^−3^
1-Stearoyl-GPC (18:0)	Lipid	Lysophospholipid	Metabolon	0.50	0.30	0.71	2.70×10^−3^
Total cholesterol to total lipids ratio in small LDL	NMR ratio/percentage	NA	Nightingale	0.44	0.24	0.63	4.08×10^−3^
3-Hydroxyoctanoate	Lipid	Fatty acid, monohydroxy	Metabolon	−0.52	−0.73	−0.30	5.14×10^−3^
Non-esterified cholesterol in small HDL	Lipid	NA	Nightingale	−0.40	−0.59	−0.22	5.24×10^−3^
Carnitine	Lipid	Carnitine metabolism	Metabolon	0.40	0.23	0.57	5.67×10^−3^
2-Docosahexaenoylglycerol (22:6)^a^	Lipid	Monoacylglycerol	Metabolon	0.55	0.32	0.79	6.05×10^−3^
γ-Glutamylglutamine	Peptide	γ-Glutamyl amino acid	Metabolon	0.45	0.25	0.64	8.94×10^−3^
Histidine	Amino acid	Histidine metabolism	Metabolon	0.50	0.28	0.72	1.07×10^−2^
Ornithine	Amino acid	Urea cycle; arginine and proline metabolism	Metabolon	0.45	0.25	0.65	1.24×10^−2^
2-Hydroxybutyrate/2-hydroxyisobutyrate	Amino acid	Glutathione metabolism	Metabolon	−0.49	−0.70	−0.27	1.40×10^−2^
1-Palmitoyl−2-arachidonoyl-GPE (16:0/20:4)^a^	Lipid	Phosphatidylethanolamine	Metabolon	−0.40	−0.58	−0.22	1.45×10^−2^
Glycerol	Lipid	NA	Nightingale	−0.48	−0.71	−0.24	1.57×10^−2^
*n*−3 Fatty acids	Lipid	NA	Nightingale	0.34	0.17	0.50	1.71×10^−2^
Aconitate (*cis* or *trans*)	Energy	TCA cycle	Metabolon	−0.45	−0.65	−0.25	2.01×10^−2^
5-Methylthioadenosine	Amino acid	Polyamine metabolism	Metabolon	−0.48	−0.69	−0.26	2.06×10^−2^
5α-Androstan−3β,17β-diol monosulfate (2)	Lipid	Androgenic steroids	Metabolon	−0.38	−0.55	−0.21	2.48×10^−2^
Cholesteryl esters in large HDL	Lipid	NA	Nightingale	0.32	0.16	0.49	2.52×10^−2^
Glycoprotein acetyls, mainly a1-acid glycoprotein	Peptide	NA	Nightingale	−0.32	−0.49	−0.16	2.57×10^−2^
Taurine	Amino acid	Methionine, cysteine, SAM and taurine metabolism	Metabolon	0.45	0.24	0.66	2.83×10^−2^
Glycine	Amino acid	Glycine, serine and threonine metabolism	Metabolon	0.40	0.22	0.59	3.07×10^−2^
Oxalate (ethanedioate)	Cofactors and vitamins	Ascorbate and aldarate metabolism	Metabolon	0.40	0.21	0.58	4.69×10^−2^

Effect estimates (β) shown for the fixed effect ‘allocation’ where control group is considered the reference group with effect estimates, therefore representing the difference seen in the intervention group relative to the control group (in normalised SD units). For additional metadata see ESM Tables 2, 3, 6

^a^Compound has not been confirmed based on a standard

CMPF, 3-carboxy-4-methyl-5-propyl-2-furanpropanoic acid; GPC, glycerophosphocholine; GPE, glycerophosphoethanolamine; NA, not applicable; SAM, S-adenosylmethionine

#### Comparison of intervention and incident type 2 diabetes footprints

**Fig. 3:**
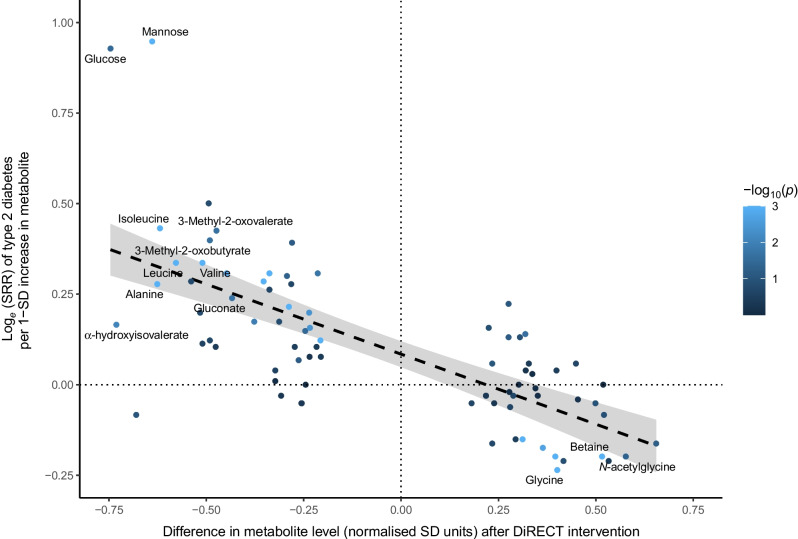
Comparison of intervention and incident type 2 diabetes footprints. Log_*e*_ SRR estimates extracted from incident type 2 diabetes meta-analysis [5] plotted against mean intervention effects (βs) from our linear regression models (these βs represent the mean difference in metabolite levels at 12 months in the intervention group relative to the control group). Each point represents a metabolite that was associated with intervention in the primary analysis (unadjusted *p*<0.05) and could be matched to meta-analysis results; metabolites with Holm-corrected *p*<0.05 in the primary analysis are labelled. Points are coloured according to the SRR-associated *p* value in the meta-analysis. The correlation is −0.70 (95% CI −0.80, −0.57, *p*=6.0×10^−13^). See ESM Fig. 8 for results including all matched metabolites

Of 622 unique metabolites with (unadjusted) *p*<0.05 in the primary analysis, 79 were matched to entries in the meta-analysis results [5], including 13 that passed the threshold for association in the primary analysis (Holm-corrected *p*<0.05). In this subset of intervention-associated metabolites, the correlation between the intervention effect βs and the log_*e*_ SRR of incident type 2 diabetes extracted from the meta-analysis was −0.70 (95% CI −0.80, −0.57, *p*=6.0×10^−13^) (Fig. 3. When comparing estimates across all 143 matched metabolites, the correlation was −0.61 (95% CI −0.71, −0.50, *p*=4.9×10^−16^)(ESM Fig. 8, ESM Table 8).

### Effect of intervention on metabolites: logistic regression model

Data from the logistic model (based on the PA dataset and able to detect and describe the presence of xenobiotics) formed the primary result for 190 of the MS metabolites, most of which were classified as xenobiotics or unidentified molecules (Table 3 and ESM Table 9). Of these, 19 (12 identified) were associated with the intervention (*p*<0.05), with 11 (8 identified) showing depletion in the intervention group. Metformin showed the strongest association and was present in 26% (31/117) of the 12 month samples from those in the intervention group compared with 78% (112/144) in the control group (see also ESM Results).

**Table 3:** Metabolites associated with intervention from logistics model (named/annotated features only)

Biochemical name	Super pathway	Sub-pathway	β	Lower 95% CI	Upper 95% CI	*p* value	State in intervention group
Metformin	Xenobiotics	Drug-metabolic	−3.45	−4.37	−2.54	1.45×10^−13^	Depleted
I-Urobilinogen	Cofactors and vitamins	Haemoglobin and porphyrin metabolism	−0.93	−1.49	−0.36	1.26×10^−3^	Depleted
β-Guanidinopropanoate	Xenobiotics	Food component/plant	−0.97	−1.56	−0.37	1.50×10^−3^	Depleted
Bradykinin, des-Arg(9)	Peptide	Polypeptide	−0.99	−1.61	−0.37	1.82×10^−3^	Depleted
3-Hydroxyindolin−2-one sulfate	Xenobiotics	Food component/plant	−0.87	−1.44	−0.30	2.80×10^−3^	Depleted
Imidazole propionate	Amino acid	Histidine metabolism	−0.86	−1.43	−0.29	2.97×10^−3^	Depleted
4-Ethylphenol glucuronide	Xenobiotics	Food component/plant	1.30	0.39	2.21	5.31×10^−3^	Enriched
S-Allylcysteine	Xenobiotics	Food component/plant	0.72	0.21	1.23	5.38×10^−3^	Enriched
Bendroflumethiazide	Xenobiotics	Drug-cardiovascular	−3.01	−5.2	−0.83	6.79×10^−3^	Depleted
Ethyl glucuronide	Xenobiotics	Chemical	−0.91	−1.58	−0.24	7.46×10^−3^	Depleted
Eicosapentaenoylcholine	Lipid	Fatty acid metabolism (acyl choline)	0.71	0.18	1.24	8.30×10^−3^	Enriched
12,13-DiHOME	Lipid	Fatty acid, dihydroxy	0.66	0.17	1.16	8.88×10^−3^	Enriched

Effect estimates (βs) are given for the fixed effect ‘allocation’ and represent the coefficient for intervention group (i.e. the log_*e*_ OR between intervention group and control group). For results in full, see ESM Table 9

12,13-DiHOME, 12,13-dihydroxy-9Z-octadecenoic acid

## Discussion

We observed a broad metabolomic signature associated with the intervention, with differences observed in metabolites from every one of the biochemical classes (or super pathways) represented. Under a conservative correction for multiple testing, 26% of NMR-derived metabolites and 12% of MS-derived metabolites were altered by the intervention, suggesting that a sizeable proportion of metabolite changes in type 2 diabetes are modifiable. We report a lipid pattern change with reduction in TG-rich lipoproteins across the lipoprotein cascade, but enrichment of (lyso)plasmalogens and reversal of amino acid changes associated with type 2 diabetes, as well as a reduction in a range of sugars beyond glucose, including fructose and mannose.

A major strength of this study is the use of samples and clinical data collected from a relatively large (compared with existing literature) cluster randomised trial with a well-matched control arm. Measuring metabolites both at baseline and at 12 months added to the robustness of the analysis while the use of two complementary metabolomics platforms increased the overall coverage of the metabolome beyond that which has been evaluated previously. However, the MS data are semi-quantitative meaning that these findings require further validation using targeted techniques to allow absolute quantification. Twelve-month samples were not available from participants who dropped out of the trial; this was only a small number and since the primary analyses concerned paired baseline and 12 month measures, bias from this differential missingness was minimised. While our study design enabled us to conduct a thorough evaluation of the metabolomic impact of the Counterweight-Plus intervention overall, it is challenging to attribute those changes to specific elements of the intervention (e.g. to fat loss per se or ‘upstream’ changes in diet). Results from attempts here to extract the weight-loss effects should be interpreted with caution, especially given that these analytical manoeuvres alter the trial structure and, as such, have the potential to introduce bias.

### Characteristic changes in metabolite profile

Many of the metabolites influenced by the intervention have also been identified as potential risk factors for type 2 diabetes development with opposite and proportionate effect sizes. For example, we see decreased concentrations of BCAAs following dietary intervention where plasma concentrations of these BCAAs are frequently elevated in type 2 diabetes [5, 9, 21]. The decrease we observe in BCAAs largely agrees with existing findings from smaller, targeted studies of weight-loss interventions [14, 22, 23]. While not all studies report this decrease in BCAAs after diet-induced weight loss [24], this may be due to a lack of power to discern what is likely to be a smaller effect than that from equivalent analyses considering surgical interventions. By using an untargeted metabolome-wide approach, in this work we were able to further characterise the plasticity of this highly relevant network. We observed concurrent decreases in several γ-glutamyl BCAA dipeptides allocated to the same cluster as the BCAAs themselves; γ-glutamyl amino acids are produced when the enzyme, γ-glutamyl transpeptidase, present mainly in the liver, catalyses the transfer of the γ-glutamyl moiety of glutathione to an amino acid [25].

Similarly to BCAAs, the reduction we see in the levels of several simple sugars, including the monosaccharides fructose, glucose and mannose, are opposite to the elevations seen in levels of these metabolites in the presence of obesity. While structurally similar, the predominant dietary sources, metabolic pathways and biological effects of these simple sugars are quite different though interdependent [26]. Mannose specifically has been associated not only with insulin resistance but also with higher risk of several chronic diseases including type 2 diabetes and CVD [27]. The existing literature concerning the most strongly associated metabolite from the MS dataset, erythronate, is limited; however, further insight into its relevance to type 2 diabetes can be gained by considering its relationship with other measured metabolites. In our data, erythronate sits in a cluster with (i.e. is correlated with) several common sugar alcohols (ribitol, erythritol, arabitol/xylitol) that can be found naturally in fruits but that are also commonly used as artificial sweeteners. Erythritol is predominantly excreted in urine, with the remaining 5–10% being oxidised to erythronate [28]. While designated as a xenobiotic in the Metabolon data and previously thought not to be produced endogenously [29], erythritol may in fact be produced endogenously from glucose [30]. This, together with preliminary evidence showing an association between erythritol and adiposity gain in young adults [30], serves to contextualise our findings of reduced levels of erythronate and related metabolites (ribitol, orotidine and erythritol all had *p*<0.05 before Holm-correction) in participants in the intervention group.

Participants in the intervention arm saw increases in several lipids previously associated with a favourable metabolic profile. Specifically, increases were seen in concentrations of several (lyso)plasmalogens, a special class of phospholipids characterised by the presence of a vinyl–ether bond at the *sn*-1 position. In a cross-sectional study of participants with overweight and obesity, plasmalogen levels were found to be inversely correlated with body fat percentage but seemingly not related to BMI or WHR [31]. The lack of association for these commonly used indicators of adiposity may be related to their suboptimal performance as proxies for adiposity in this relatively small sample of individuals all with BMI>25 kg/m^2^ (*n*=65). Alternatively, this may point towards a more complex interplay between metabolic health and plasmalogens.

### Detecting associations with changes in exogenous factors

While changes to the metabolism can be expected in response to the intervention-induced weight loss experienced by many of those in the intervention group, we also expect the adoption of new dietary patterns and a change in medication regimes. At a metabolomic level, and notwithstanding possible limitations linked to limits of detection, we assume that, where we see high levels of missingness for given metabolites, these patterns are indicative of absence and/or very low concentration. This does mean that naive application of a linear model will be underpowered to detect mean differences in concentrations. However, examining between-group differences in presence/absence can allow detection of meaningful relationships. To this end, the logistic regression analysis here revealed between-group differences in the frequency of detection of both potential dietary biomarkers and medications. For example, *S*-allylcysteine, a proposed biomarker for garlic consumption [32], was enriched in the intervention group while ethyl glucuronide, a validated urine biomarker for alcohol consumption [33, 34] was depleted. The reduced presence of metformin in the intervention group at 12 months provides a useful positive control as well as offering an opportunity to verify medication usage. In the linear regression analysis, the association of omega-3 (*n*-3) fatty acids with allocation did not attenuate with additional adjustment for weight change. This suggests that the increased levels of these essential fats in participants from the intervention arm are due to dietary changes (sustained at 12 months) and are not directly related to weight loss.

### Metabolite profile variation and clinically relevant biomarkers

Metabolic profile at 12 months, as captured by the intervention-associated metabolites, was strongly correlated with weight change, possibly explaining much of the allocation effect that we observe. Indeed, the changes we see in levels of glucose and BCAAs are characteristic of those seen with weight change in other settings [20, 35, 36]. We see a decrease in the TG/total lipids ratio across LDLs and VLDLs in participants in the intervention group, with what appears to be a corresponding increase in the total cholesterol and/or cholesteryl ester/total lipids ratio in a similar subset of lipoproteins as would be expected given the previously characterised decrease in hepatic production of VLDL TG following dietary weight loss in type 2 diabetes [37]. These effects are in keeping with the proposed mechanism by which excess TG in the circulation triggers the transfer of TGs from the core of TG-rich lipoproteins to LDL in exchange for cholesteryl esters by the cholesteryl ester transfer protein [38].

The metabolic profile at 12 months was also correlated with change in HbA_1c_, demonstrating the ability of the intervention-associated metabolites identified to capture changes in glycaemic health as expressed by traditional clinical biomarkers. However, we also found evidence of subtle differences in the metabolome of those who achieved type 2 diabetes remission as compared with those that did not despite similar levels of weight loss. For example, among those individuals who lost the most weight (greater than 9.0 kg) during the trial, those who also achieved diabetes remission showed greater decreases in glucose, fructose and mannose, as compared with those who did not achieve remission. To some extent, this likely reflects the diagnostic criteria on which remission status was based; indeed, 1,5-anhydroglucitol (a proposed marker of short-term glycaemic control [39]) also appears in the list of metabolites that showed differential change by remission status. Based on the current analysis, we are unable to determine whether these differences reflect metabolic processes that contribute to remission or are simply a reflection of an individual’s current metabolic health status. Meanwhile, the correlations observed between intervention-associated changes in metabolites and clinical indicators of liver health suggest that by conducting an in-depth analysis of metabolites in the presence of sustained improvements to liver health, as here, we can further investigate proposed biological systems, such as the twin cycle hypothesis [40], including in the context of variable individual response (see ESM Discussion).

## Conclusion

In conclusion, we have characterised the impact of weight loss in type 2 diabetes at the level of the metabolome. The changes we observed were evident many weeks after the conclusion of the weight-loss phase of the intervention, indicating sustained benefits to health. Our results suggest that previously described perturbations of metabolite profile in incident type 2 diabetes are reversible with intentional weight loss while there is little evidence for any obvious adverse metabolic signals. The extent to which an individual’s metabolic profile is normalised relative to the level seen in healthy control individuals requires further research. Of the clinical variables tested, weight change was most strongly correlated with the overall change in metabolic profile associated with the intervention. This suggests that weight change is upstream of many disease-associated metabolite alterations, in line with growing consensus of the importance of excess adiposity in the pathogenesis of diabetes and, as a treatment target, as reflected in recent ADA/EASD recommendations [41]. Use of data from an RCT of a clinically proven dietary intervention now adopted by the NHS for patients with type 2 diabetes makes these results both generalisable to the patient population and highly clinically relevant. Validation of our findings in larger studies and, in the case of MS data, the use of methods that allow absolute quantification is warranted. This work provides an opportunity for detailed comparisons of different weight-loss interventions (beyond weight and basic measures) in the future, including consideration of the multiple newly emerging pharmacological therapies.

### Supplementary Information

Below is the link to the electronic supplementary material.

Supplementary file1 (PDF 489 KB)

Supplementary file2 (XLSX 530 KB)

## Data Availability

The data used for analysis are available on a research data repository (https://researchdata.gla.ac.uk/) with access given to researchers subject to appropriate data sharing agreements. Requests to access data should be made to the Principal Investigators of DiRECT via https://www.directclinicaltrial.org.uk/directstudyteam.html quoting project title ‘What lies behind the causal impact of body mass index (BMI) level and change on human health? Added value from complementary study design and deep metabolic phenotyping’. Metabolite data preparation, data pre-processing, statistical analyses and figure generation were performed in R Studio v.1.0.143 [42] using R v.4.0.2 [43]. The R code for this study has been made publicly available on GitHub at: https://github.com/lauracorbin/metabolomics_of_direct.

## Abbreviations

BCAA: Branched-chain amino acid
DiRECT: Diabetes Remission Clinical Trial
NHS: National Health Service
PA: Dataset with data transformed to a presence/absence phenotype with missing values replaced with 0 and non-missing values with 1
PC: Principal component
PCA: Principal component analysis
QC: Quality control
RNT: Dataset with data transformed using a rank-based inverse normal transformation
SRR: Summary relative risk
TG: Triglyceride
