# Supplementary material for: The metabolomic signature of weight loss and remission in the Diabetes Remission Clinical Trial (DiRECT)
Source: Diabetologia. 2023 Oct 25;67(1):74–87. doi: 10.1007/s00125-023-06019-x (PMC10709482; doi:10.1007/s00125-023-06019-x)
Supplement: Supplementary file 1 — Supplementary file1 (PDF 489 KB) [file 125_2023_6019_MOESM1_ESM.pdf]

Electronic Supplemental Material (ESM) for:

The metabolomic signature of weight loss and remission in the Diabetes Remission Clinical Trial (DiRECT)

Corbin *et al.*

Table of Contents

ESM Methods *4*

Sample collection and metabolite data acquisition **4**

Sample collection 4

Nuclear magnetic resonance spectroscopy 4

Mass spectrometry data 4

Metabolite data preparation **6**

NMR data 6

MS data 7

Statistical analysis **8**

Linear regression model 8

Exploratory analysis of associated metabolites and clinical phenotypes 8

Figure generation **10**

ESM Results *10*

Exploratory analysis of associated metabolites and clinical phenotypes **10**

Effect of intervention on metabolites – logistic regression model **11**

ESM Discussion *11*

Exploratory analysis of associated metabolites and clinical phenotypes **11**

ESM Tables *12*

ESM Table 1 Overview of metabolite data post-curation filtering **12**

ESM Table 2 NMR data: Results of linear model **12**

ESM Table 3 MS data: Results of linear model **12**

ESM Table 4 Metabolite change in individuals who did and did not achieve diabetes remission within quantiles of weight change (associated metabolites only) **12**

ESM Table 5 Super pathway and cluster allocation **12**

ESM Table 6 Associated representative metabolites from linear model (restricted to named/annotated features only) **12**

ESM Table 7 Relationship between the derived (top) PCs and the select clinical phenotypes as assessed by Pearson's correlation (r) **13**

ESM Table 8 Comparison of intervention and incident type 2 diabetes footprints **13**

ESM Table 9 MS data: Results of logistic model **13**

ESM Figures *14*

ESM Figure 1. A demonstration of the poor performance of the linear model below the chosen threshold of missingness (<40%) **14**

ESM Figure 2. Association of metabolites run in linear model with intervention status. **15**

ESM Figure 3. Distribution of metabolites across super pathways. **16**

ESM Figure 4. Change in metabolite summarised in weight change categories and by remission status **17**

ESM Figure 5. Scree from PCA performed on data for the 61 associated representative metabolites **19**

ESM Figure 6A. Principal component analysis (PCA) on samples using data for 61 associated representative metabolites after adjustment for covariates: biplot of general metabolic health. 20
ESM Figure 6B. Principal component analysis (PCA) on samples using data for 61 associated representative metabolites after adjustment for covariates: biplot of NAFLD. 21
ESM Figure 7. Principal component analysis (PCA) on samples using data for 61 associated representative metabolites after adjustment for covariates: loadings plot. 22
ESM Figure 8. Comparison of intervention and incident type 2 diabetes footprints including all matched metabolites. 23
References 24

ESM Methods

Sample collection and metabolite data acquisition

Sample collection

Blood for the serum samples was drawn into an SST serum separator tube then kept at 4°C until being processed. Within five hours of blood draw samples were centrifuged at 2000g for 15 minutes at 4°C then the serum was separated and stored as five 0.5 mL aliquots at -80°C. Samples collected in Scotland were transported as whole blood at 4°C to the Central Glasgow laboratory (BHF GCRC, University of Glasgow) and processed there, whilst samples collected in the Newcastle area were processed locally and stored as serum at -80°C before transfer to Glasgow.

Aliquots run on the two different metabolomics platforms were extracted from the same sample source. Samples were sent first to Metabolon, Inc. (Durham, North Carolina, USA) where they were thawed for the first time for aliquoting. Remaining sample material was then re-frozen and sent to the MRC Integrative Epidemiology Unit Metabolomics Facility (University of Bristol) for ^{1}H-NMR analyses (after one further thaw).

Nuclear magnetic resonance spectroscopy

A serum ^{1}H-NMR metabolomics platform (Nightingale Health Ltd, Helsinki, Finland) was used to quantify circulating metabolites with an emphasis on lipid or lipoprotein lipid measures. Details of the experimentation have been described elsewhere [1, 2]. This high-throughput metabolomics platform provides simultaneous quantification of routine lipids, lipid concentrations of 14 lipoprotein subclasses and major subfractions, and further abundant fatty acids, amino acids, ketone bodies, and gluconeogenesis-related metabolites. The measured variables include 148 primary measures quantified in absolute concentrations as well as 79 additional derived measures such as ratios and percentages, primarily related to fatty acids and lipoprotein composition. Herein, this dataset is referred to as 'NMR data' and the 79 additional measures specifically referred to as 'derived measures.' Data were received from Nightingale on 19th March 2019.

Mass spectrometry data

An untargeted metabolomics analysis of metabolites was performed at Metabolon, Inc. (Durham, North Carolina, USA) using established protocols, as described in published work [3, 4]. In brief, the Metabolon analysis consisted of four independent ultra-high-performance liquid chromatography-tandem mass spectrometry (UPLC-MS/MS) runs. All methods utilized a Waters ACQUITY ultra-performance liquid chromatography (UPLC) and a Thermo Scientific Q-Exactive high resolution/accurate mass spectrometer interfaced with a heated electrospray ionization (HESI-II) source and Orbitrap mass analyzer operated at 35,000 mass resolution. Raw data were extracted, peak-identified and quality control (QC) processed using Metabolon's hardware and software. Compounds were identified by comparison to library entries of purified standards or recurrent unknown entities. Further details (as provided by Metabolon, Inc.) can be found below. Baseline and 12-month samples from the same individuals were analysed in the same batch to avoid the confounding of technical variation (batch effects) with time point. The resulting dataset

comprised a total of 1,276 metabolite features comprising 959 compounds of known identity (named biochemicals with the majority matched to purified standards) and 317 compounds of unknown structural identity (unnamed biochemicals) as of February 2018 when data were generated (subsequent library updates are described in below). Herein, this dataset is referred to as 'MS data.' Data were issued by Metabolon on 16th February 2018.

Sample preparation

Samples were prepared using the automated MicroLab STAR® system from Hamilton Company. Several recovery standards were added prior to the first step in the extraction process for QC purposes. Proteins were precipitated with methanol under vigorous shaking for 2 min (Glen Mills GenoGrinder 2000) followed by centrifugation. The resulting extract was divided into five fractions: two for analysis by two separate reverse phase (RP)/UPLC-MS/MS methods with positive ion mode electrospray ionization (ESI), one for analysis by RP/UPLC-MS/MS with negative ion mode ESI, one for analysis by HILIC/UPLC-MS/MS with negative ion mode ESI, and one for backup. Samples were placed briefly on a TurboVap® (Zymark) to remove the organic solvent. The sample extracts were stored overnight under nitrogen before preparation for analysis.

Quality assurance / Quality control

Three types of controls were used when analyzing the experimental samples: a pooled matrix sample generated by taking a small volume of each experimental sample (or alternatively, use of a pool of well-characterized human plasma); extracted water samples; and a cocktail of QC standards. Instrument variability was determined by calculating the median relative standard deviation (RSD) for the standards that were added to each sample prior to injection into the mass spectrometers. Overall process variability was determined by calculating the median RSD for all endogenous metabolites (i.e., non-instrument standards) present in 100% of the pooled matrix samples. Experimental samples were randomized across the platform run with QC samples spaced evenly among the injections.

Ultrahigh Performance Liquid Chromatography-Tandem Mass Spectroscopy (UPLC-MS/MS)

All methods utilized a Waters ACQUITY ultra-performance liquid chromatography (UPLC) and a Thermo Scientific Q-Exactive high resolution/accurate mass spectrometer interfaced with a heated electrospray ionization (HESI-II) source and Orbitrap mass analyzer operated at 35,000 mass resolution. The sample extract was dried then reconstituted in solvents compatible to each of the four methods. Each reconstitution solvent contained a series of standards at fixed concentrations to ensure injection and chromatographic consistency. One aliquot was analyzed using acidic positive ion conditions, chromatographically optimized for more hydrophilic compounds. In this method, the extract was gradient eluted from a C18 column (Waters UPLC BEH C18-2.1x100 mm, 1.7 µm) using water and methanol, containing 0.05% perfluoropentanoic acid (PFPA) and 0.1% formic acid (FA). Another aliquot was also analyzed using acidic positive ion conditions, however it was chromatographically optimized for more hydrophobic compounds. In this method, the extract was gradient eluted from the same afore mentioned C18 column using methanol, acetonitrile, water, 0.05% PFPA and 0.01% FA and was operated at an overall higher organic content. Another aliquot was analyzed using basic negative ion optimized conditions using a separate dedicated C18 column. The basic extracts were gradient eluted from the column using methanol and water, however with 6.5mM Ammonium Bicarbonate at pH 8. The

fourth aliquot was analyzed via negative ionization following elution from a HILIC column (Waters UPLC BEH Amide 2.1x150 mm, 1.7 µm) using a gradient consisting of water and acetonitrile with 10mM Ammonium Formate, pH 10.8. The MS analysis alternated between MS and data-dependent MSn scans using dynamic exclusion. The scan range varied slighted between methods but covered 70-1000 m/z.

Data extraction and compound identification

Raw data were extracted, peak-identified and QC processed using Metabolon's hardware and software. Compounds were identified by comparison to library entries of purified standards or recurrent unknown entities. More than 3300 commercially available purified standard compounds have been acquired and registered into LIMS for analysis on all platforms for determination of their analytical characteristics. Additional mass spectral entries have been created for structurally unnamed biochemicals, which have been identified by virtue of their recurrent nature (both chromatographic and mass spectral).

Metabolite Quantification and Data Normalization

Peaks were quantified using area-under-the-curve. A data normalization step was performed to correct variation resulting from instrument inter-day tuning differences.

Metabolite library updates

In February 2018, data for 959 known and 317 unnamed biochemicals were returned by Metabolon. In January 2022, Metabolon issued revised identifications for the following metabolites:

Incorrect identification	Correct identification
1-carboxyethylleucine	N-lactoyl leucine
1-carboxyethylisoleucine	N-lactoyl isoleucine
1-carboxyethylphenylalanine	N-lactoyl phenylalanine
1-carboxyethyltyrosine	N-lactoyl tyrosine
1-carboxyethylvaline	N-lactoyl valine
1-carboxyethylhistidine	N-lactoyl histidine

Metabolite data preparation

A series of data quality checks were carried out on each metabolomics dataset using a pre-release version of the R package *metaboprep* [5] with samples and features excluded from subsequent statistical analysis based on a pre-defined set of QC metrics.

NMR data

Missingness was assessed by samples and by metabolite feature. By sample missingness rates were calculated based on the 148 primary measures (i.e. excluding derived measures[1]). Three samples with more than 20% of the primary measures unquantified were

[1] In data from Nightingale Health, derived variables are metabolite traits that are a summary of two or more other metabolites (possibly already represented in the dataset). These variables can introduce bias in estimates of sample missingness (where a single metabolite is missing, any derived measures based on that

excluded. By feature missingness was calculated for all 227 measures after sample exclusions had been applied and any features with >20% missing values flagged for exclusion. The only primary feature to be excluded was 22:6, docosahexaenoic acid (DHA), concentrations of which were returned for only 32.2% of samples. This was likely due to interference with the quantification of this metabolite in most of the samples, probably originating from the sample tubes used for sample collection. Therefore, measures of DHA and the ratio of DHA to total fatty acids were excluded from all analyses. After these sample and feature exclusions, the median (minimum, maximum) rate of sample missingness in primary measures (i.e. excluding derived measures) was 0.00 (0.0, 1.4) % and the median rate of metabolite feature missingness (across all 225 measures) was 0.0 (0.0, 14.5) %.

Following sample and feature filtering based on missingness, a principal component analysis (PCA) was performed (using the 147 primary measures only) to identify potential sample outliers. First, a hierarchical clustering approach was used to reduce the redundancy in the data (after exclusion of the derived measures). A Spearman's correlation matrix was generated using the 'pairwise complete observations' of the base R cor() function applied to metabolite data for the 147 primary measures. From this a distance matrix was constructed (calculated as 1 minus the absolute correlations) and used to build a dendrogram using hclust() and the method 'complete' from the R 'stats' package. The resulting tree was cut at a height of 0.2 (corresponding to a maximum within cluster correlation of 0.8) giving 37 groups of correlated metabolites. A single representative metabolite was selected from each group for inclusion in the PCA based on minimum missingness. This reduced dataset was then centred and scaled, and a probabilistic PCA [6] implemented; the probabilistic method was used to allow for missing values. Samples located more than five standard deviations (5 SD) from the mean of the first and/or second principal components were excluded. The PCA was then re-run to check for further outliers. This procedure resulted in the exclusion of one sample. Finally, three samples were removed based on quality control tags supplied as part of the data release supplied by Nightingale and indicative of poor sample quality, namely, high pyruvate, high lactate and low glutamine/high lactate ratio (as compared to the expected values based on benchmarking). After filtering, there were 567 samples and 225 metabolites (147 primary measures and 78 derived ratios) available for the next steps in the analysis.

MS data

Missingness was assessed by samples and by metabolite feature. By sample missingness rates were calculated after excluding 203 features designated as xenobiotics[2] (leaving 1073 metabolites). There were no samples with more than 20% of metabolites unquantified and therefore no samples were excluded based on high missingness. By feature missingness was then calculated for all 1,276 metabolites and 22 features with less than five observations across the entire set of samples excluded. After these sample and feature exclusions, the median (minimum, maximum) rate of sample missingness in primary measures was 15.3

metabolite will also be missing) and may not be appropriate to retain when identifying a set of representative metabolites for the data set.

[2] Xenobiotics are metabolites not produced by the body, such as drug compounds and consequently can have very high rates of missingness, while still being critically informative to a study. For this reason, we do not include xenobiotics when calculating by sample missingness but would advocate affording them special consideration in any downstream statistical analyses.

(10.3, 25.0) % and the median rate of metabolite feature missingness (across all ~~225~~ measures) was 1.0 (0.0, 99.1) %. Note, the maximum by sample missingness in the filtered set is greater than the 20% threshold applied because data for xenobiotics was included in this summary.

Following sample and feature filtering based on missingness, total peak area (TPA) per sample was calculated as the sum of peak areas across all features. Samples with a TPA more than 5 SD from the mean (above or below) were identified for exclusion; no samples were excluded based on these criteria. Next, the hierarchical clustering and probabilistic PCA was performed as described above (in the NMR data section) but using only those features with <20% missing data as input to the hierarchical clustering step. No sample outliers were identified because of the PCA. Since the same sample material was used for both the NMR and MS-based analyses, any samples excluded from the NMR data based on NMR quality control tags were also excluded from the MS dataset. After filtering, there were 571 samples and 1,254 metabolites for the next steps in the analysis.

The metabolite data preparation procedures described were performed in R Studio v.1.0.143 [7] using R v.4.0.2 [8].

Statistical analysis

Linear regression model

The variance explained in the metabolite levels (metabolite$_{T1}$) by each of the fixed effects was also derived from the model by estimating eta-squared derived from the sums of squares of a Type II ANOVA ('car' package in R). Post-hoc checks for a difference in metabolite levels at baseline were conducted for intervention-associated metabolites. First, raw metabolite data were centred (by subtracting the mean) and scaled (by dividing by the standard deviation) within time point. Then, a two-sample Wilcoxon rank sum test was performed to compare metabolite levels in control and intervention groups at baseline and at 12-months. No adjustments were made for other variables.

Exploratory analysis of associated metabolites and clinical phenotypes

For the subset of intervention-associated metabolites (as identified in the primary linear regression analysis), several follow-up analyses were performed.

To evaluate the extent to which the association between metabolite$_{T1}$ and allocation could alternatively be explained by weight change, the primary model was re-fitted with the addition of 'weight change' as a fixed effect, where weight change (kg) was calculated as weight (kg) at 12 months minus weight (kg) at baseline. The variance of metabolite$_{T1}$ explained by allocation before and after additional adjustment for weight change was compared and a percentage change calculated.

To begin to understand the potential relevance of metabolite levels to diabetes remission, over and above their role as proxies for weight loss, metabolite change was compared in individuals who did and did not achieve diabetes remission within quantiles of weight change. Metabolite change was calculated as metabolite at 12 months, minus metabolite at

baseline. The input data for this derivation was RNT metabolite data but unlike in the primary analysis, the RNT was performed such that baseline and 12-month measures were transformed together. Weight change (kg) was calculated as weight (kg) at 12 months, minus weight (kg) at baseline. Within the bottom two quantiles of weight change (i.e., in those that had lost the most weight), metabolite change was compared between patients who did or did not achieve diabetes remission using a two-sample two-sided Students t-test (assuming unequal variance). The correlation between weight change and metabolite change was also calculated within weight change quantiles.

Next, a principal component analysis (PCA)-based investigation was conducted into the relationship between the metabolites found to be associated with the intervention and the change in a subset of clinical phenotypes selected based on their relevance to the long-term health of patients with type 2 diabetes. First, data from the two platforms (NMR and MS) were combined and restricted to features with <40% missing data at 12-months (i.e., those metabolites for which the linear model represented the primary analysis). A hierarchical clustering approach (by the same method as described above in '**Metabolite data preparation**') was applied to residuals extracted from a re-run of the linear model as described in the main manuscript (using the RNT metabolite data) but without allocation (or weight change) fitted. These residuals represent the metabolite levels at 12 months after adjusting for covariates. The resulting dendrogram was cut at a height of 0.80 (which corresponds to a maximum within cluster correlation of 0.2) to define a set of metabolite clusters. The list of associated features from the linear model analysis was then reduced such that a single feature per cluster was retained for the next step; the feature to be retained was selected based on a principal variables analysis (PVA) such that the feature that captured the most variation within the cluster was kept as the representative metabolite (using 'PVA' function from the 'growthPheno' R package [9]). Model residuals for the subset of representative metabolites were then used as input to a sample based probabilistic PCA. We took this data (metabolite) reduction approach to avoid the overrepresentation specific groups of metabolites in the PCA that might otherwise occur due to the bias of the metabolomic panels towards certain classes of metabolite.

The relationship between the derived (top) PCs and a subset of clinical phenotypes selected based on their relevance to the long-term health of patients with type 2 diabetes was assessed by Pearson's correlation (r) and presented as a biplot. The clinical phenotypes included: (1) phenotypes selected for their relevance to the trial's primary outcomes: allocation (control/intervention) and type 2 diabetes remission status at 12 months where remission is defined as glycated haemoglobin ($HbA1_c$) less than 6.5% (<48 mmol/mol) after at least 2 months off all antidiabetic medications [10]; (2) a set of indicators of general metabolic health: weight (kg), glycated haemoglobin (HbA1c) (mmol/mol), diastolic and systolic blood pressure, total cholesterol (mmol/l), creatinine (umol/l), c-reactive protein (CRP) (mg/l) and quality of life as assessed by EQ-5D visual analogue scale (VAS) and utility index values; and (3) parameters relevant for non-alcoholic fatty liver disease (NAFLD), a common complication of type 2 diabetes: aspartate aminotransferase (AST) (units/l), alanine aminotransferase (ALT) (units/l), albumin-to-creatinine ratio (ACR) (mg/mmol), gamma-glutamyl transpeptidase (GGT) (units/L) and liver fat percentage.

In addition, hypergeometric-based enrichment analyses were conducted to designate a super pathway to the clusters. To do so, each cluster containing more than five metabolites was tested for enrichment for specific classes as compared to all features tested in the linear model. The cluster was then designated a super pathway annotation according to the most enriched class.

Figure generation

Heatmap (Figure 2): The heatmap was generated using the R package 'heatmap3'[11, 12]. The same set of residuals used for the PCA analysis described in the '**Exploratory analysis of associated metabolites and clinical phenotypes**' section above were used as input. Dendrograms were built for samples and metabolites using the hclust() function from the R 'stats' package with the method 'ward.D2'[13, 14] specified.

Volcano (ESM Figure 2): To enable robust statistical analyses that would not be unduly affected by distributional characteristics typical of metabolomics data such as non-normal distributions and small numbers of extreme values (typically at the high end of the distribution), the primary linear regression model-based analysis was performed on the RNT dataset. However, this can make the comparison of relative effect sizes across metabolites challenging. Therefore, we also calculated the median fold change difference on the raw data as the ratio between the two group medians (intervention group median/control group median); no adjustments were made for other variables. This fold change was transformed to a log2 scale before plotting in ESM Figure 2 (x-axis).

Biplots (ESM Figures 6A, 6B and 7): Plots presented are based on the probabilistic PCA as described in the '**Exploratory analysis of associated metabolites and clinical phenotypes**' section above and in the main manuscript. Data points (samples) in ESM Figures 6A and 6B are positioned based on scores for PC1 and PC2. Whilst the original loadings (for metabolites) are shown in ESM Figure 7, the biplot arrows in ESM Figures 6A and 6B were redefined such that the length and direction of the arrows are scaled according to the correlation coefficients between the two PCs and the selected clinical phenotypes.

ESM Results

Exploratory analysis of associated metabolites and clinical phenotypes

Prior to PCA, the set of features associated with the intervention in the linear model was reduced to a subset of approximately independent, representative features. This hierarchical clustering procedure allocated the 1,289 metabolites with <40% missing data (225 NMR and 1064 MS) to 238 metabolite clusters with a minimum intra-cluster correlation of 0.2 (**ESM Table 5**). The median cluster size was four (range: 1 to 66). Once the list of 186 associated metabolites from linear model analysis was restricted to one representative metabolite per cluster, there were 61 associated representative metabolites - 11 from the NMR platform (including two derived measures) and 50 from the MS platform, of which 10 were classified as 'unknown' by Metabolon (i.e., they were not

named/annotated) (**ESM Table 6**). Between 20% and 100% of metabolites within clusters containing an associated representative metabolite were nominally associated with intervention (unadjusted p<0.05) with the mean proportion being 72%. Of the 33 associated representative metabolites from clusters containing more than five metabolites and therefore tested for enrichment, 24 had the same super pathway designation as that for which its cluster showed enrichment.

To identify clinical phenotypes associated with intervention associated metabolite profiles we derived Pearson's correlations between metabolite derived PCs (PCA scree plot shown in **ESM Figure 5)** and change in clinical phenotypes. Of the clinical phenotypes considered as indicators of general metabolic health, the strongest correlations with PC1 were observed for change in weight (r=-0.71, 95% CI: -0.76, -0.64), p=4.1×10^{-41}), change in HbA1c (r=-0.52, 95% CI: -0.60, -0.44, p=2.2×10^{-19}) and change in EQ-5D visual analogue scale (EQ-5D VAS) (r=0.32, 95% CI: 0.20, 0.42, p=1.8×10^{-07}) (**ESM Figure 6A**, **ESM Table 7A**). The strongest correlations with PC2 (which explained 7% of the variance) were observed for change in total cholesterol (r=-0.31, 95% CI: -0.41, -0.19, p=4.8×10^{-07}) and change in creatinine (r=-0.13, 95% CI: -0.25, -0.01, p=0.03) (**ESM Figure 6A**, **ESM Table 7A**). Of the clinical phenotypes considered relevant for non-alcoholic fatty liver disease (NAFLD), a moderate negative correlation was observed between PC1 and all change phenotypes, with the strongest correlation seen for change in liver fat percentage (r=-0.62, 95% CI: -0.75, -0.44, p=7.2×10^{-08}) (**ESM Figure 6B, ESM Table 7B**). The contribution of the representative associated metabolites to the PCs is relatively consistent with their pattern of association with the intervention, such that the metabolites most strongly associated with the intervention, for example, glucose and isoleucine, have relatively large loadings for PC1 (**ESM Figure 7**).

Effect of intervention on metabolites – logistic regression model

Concordance between metformin use as indicated by General Practitioner (GP) records and the presence/absence of metformin in blood samples was high both at baseline and 12 months with at most 15 discrepant cases out of N=261 (i.e., cases where metformin was detected in samples from patients whose records indicated no prescription or vice versa).

ESM Discussion

Exploratory analysis of associated metabolites and clinical phenotypes

The moderate correlations we see between PC1 and liver fat percentage and clinical indicators of liver health (e.g., aspartate aminotransferase (AST), alanine aminotransferase (ALT)) in the exploratory PCA analysis speak to the ability of this metabolome-wide approach to capture biological signatures of the effect of weight loss on liver health previously evidenced in DiRECT [15]. Studies have shown the effect of low energy diets on the mobilization of liver fat to be almost instantaneous with dramatic decreases in liver fat seen within seven days because of the negative calorie balance and need to mobilize energy stores [16]. By conducting an in-depth analysis of metabolites in the presence of sustained improvements to liver health as here, we can further investigate proposed biological systems, such as the twin cycle hypothesis [17], including in the context of variable patient response.

ESM Tables

ESM Table 1 Overview of metabolite data post-curation filtering

		NMR data	MS data
	**ORIGINAL DATASET**	**574 samples<br>227 metabolites**	**574 samples<br>1276 metabolites**
**QC CRITERIA**	Sample missingness	Samples excluded if >20% features missing. Data from derived measures excluded.<br>**N=3 samples excluded**	Samples excluded if >20% features missing. Data from xenobiotics excluded.<br>**N=0 samples excluded**
	Feature missingness	Features excluded if >20% samples do not have a value.<br>**M=2 features excluded**	Features excluded if <5 observations.<br>**M=22 features excluded**
	Principal components analysis (PCA) to look for sample outliers	Samples excluded if >5SD from the mean on PC1 or PC2.<br>**N=1 sample excluded**	Samples excluded if >5SD from the mean on PC1 or PC2.<br>**N=0 samples excluded**
	QC fails from Nightingale indicative of poor sample quality	**N=3 samples excluded**	**N=3 samples excluded**
	**POST-FILTERING DATASET**	**567 samples<br>225 metabolites**	**571 samples<br>1254 metabolites**

ESM Table 2 NMR data: Results of linear model
See Excel file.

ESM Table 3 MS data: Results of linear model
See Excel file.

ESM Table 4 Metabolite change in individuals who did and did not achieve diabetes remission within quantiles of weight change (associated metabolites only)
See Excel file.

ESM Table 5 Super pathway and cluster allocation
See Excel file.

ESM Table 6 Associated representative metabolites from linear model (restricted to named/annotated features only)
See Excel file.

ESM Table 7 Relationship between the derived (top) PCs and the select clinical phenotypes as assessed by Pearson's correlation (r)

See Excel file.

ESM Table 8 Comparison of intervention and incident type 2 diabetes footprints

ESM Table 9 MS data: Results of logistic model

See Excel file.

ESM Figures

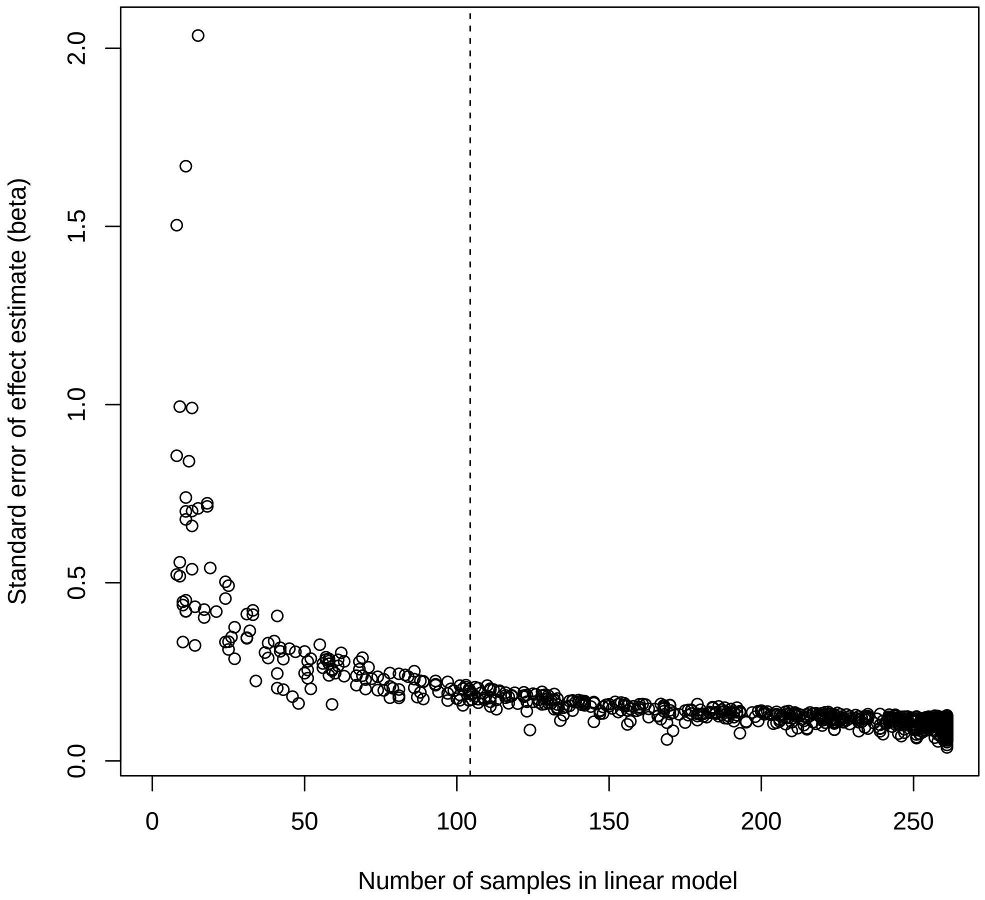

ESM Figure 1. A demonstration of the poor performance of the linear model below the chosen threshold of missingness (<40%)

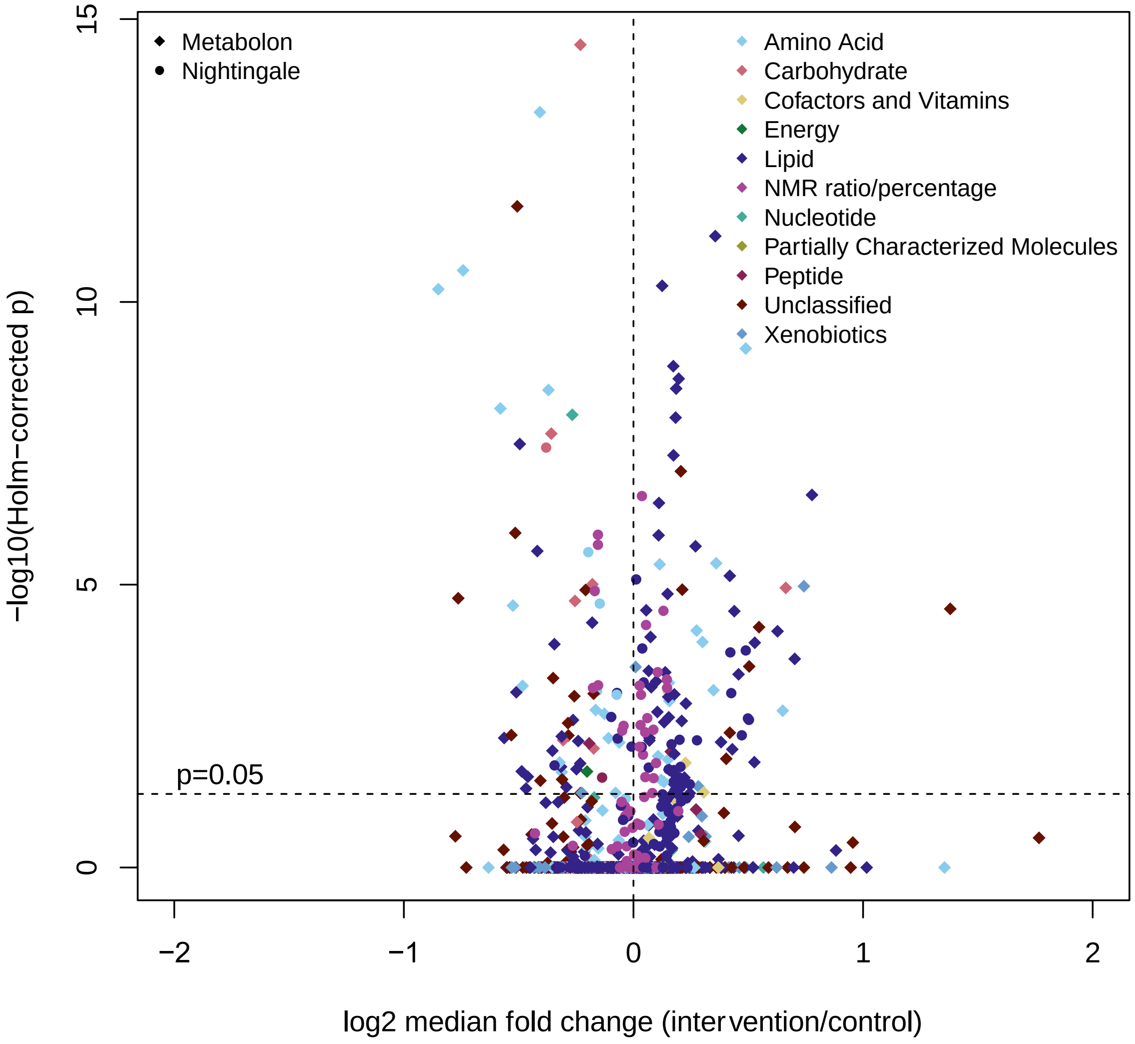

ESM Figure 2. Association of metabolites run in linear model with intervention status. Holm-corrected p-values for allocation effect extracted from linear regression model. Log_2 fold change calculated using raw (untransformed and unadjusted) metabolite data at T_1 (12 months). Horizontal dashed line indicates Holm-corrected p=0.05.

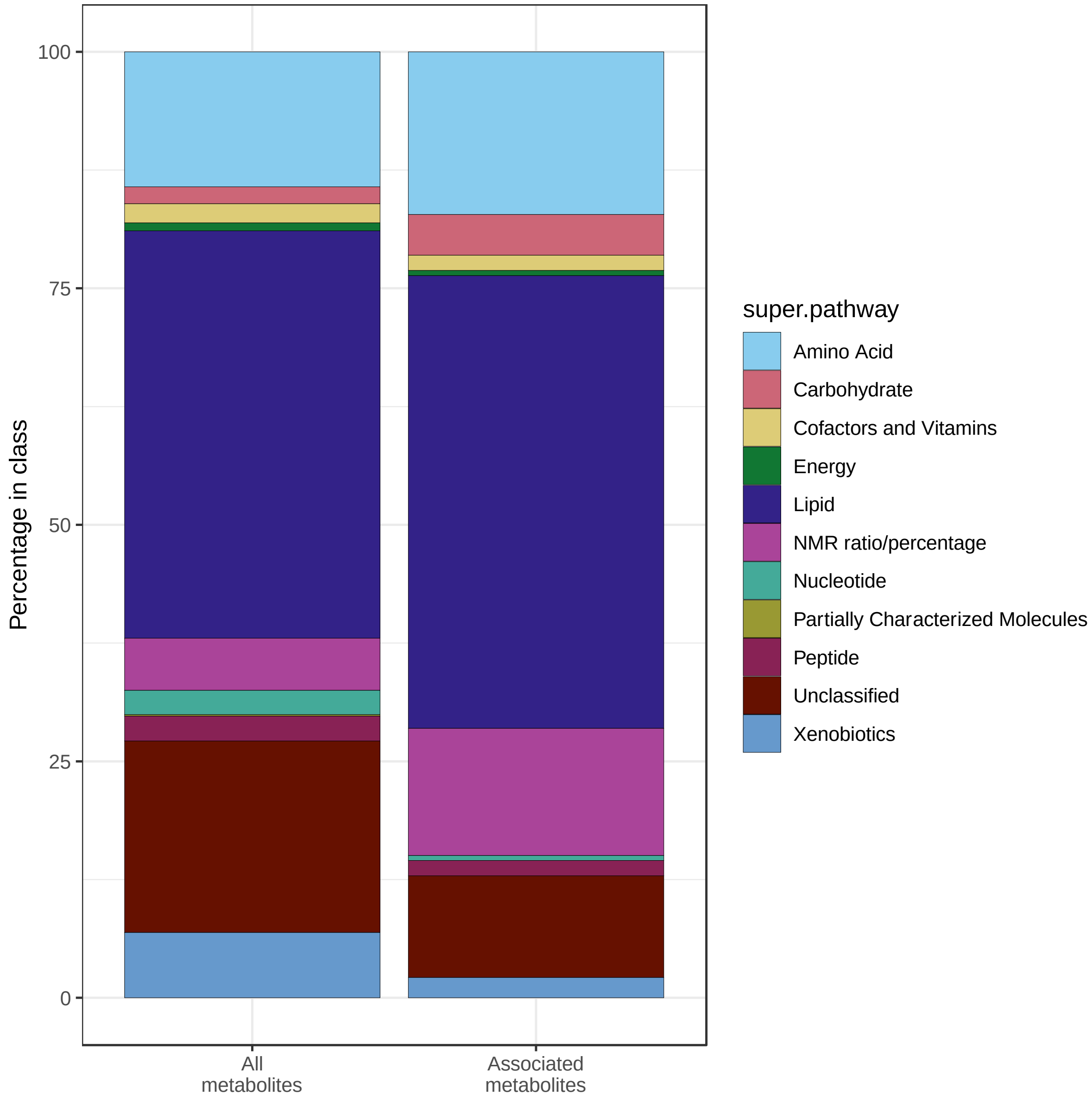

ESM Figure 3. Distribution of metabolites across super pathways.

‘All metabolites’ = all metabolites with <40% missing data (N=1289); ‘Associated metabolites’ = all associated metabolites from linear model (Holm-corrected p<0.05) (N=186). Hypergeometric-based enrichment analyses (conducted jointly across the NMR and MS datasets) gave evidence for enrichment in the associated metabolites for NMR derived measures (2.4-fold, p=5.08 x 10^{-06}) and for the carbohydrate super pathways (2.4-fold, p=0.011).

ESM Figure 4. Change in metabolite summarised in weight change categories and by remission status.

Weight change categories defined as quantiles of weight change (Q1 to Q4) with weight change range for each quantile provided. P-values presented for a two-sample two-sided Students t-test (assuming unequal variance) to test for a mean difference in metabolite change by remission status (as performed in weight change quantiles Q1 and Q2).

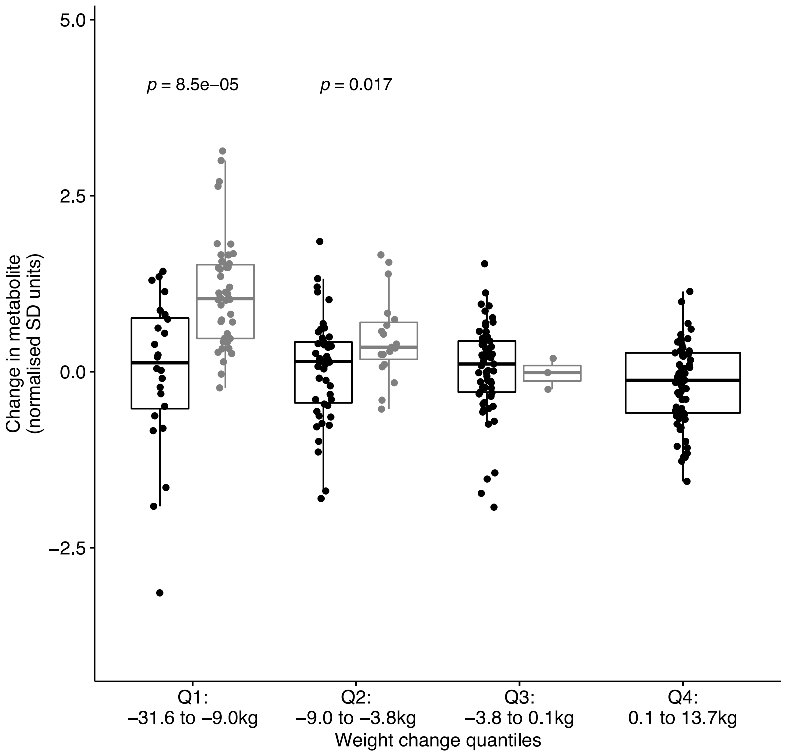

(a) 1,5-anhydroglucitol (1,5-AG)

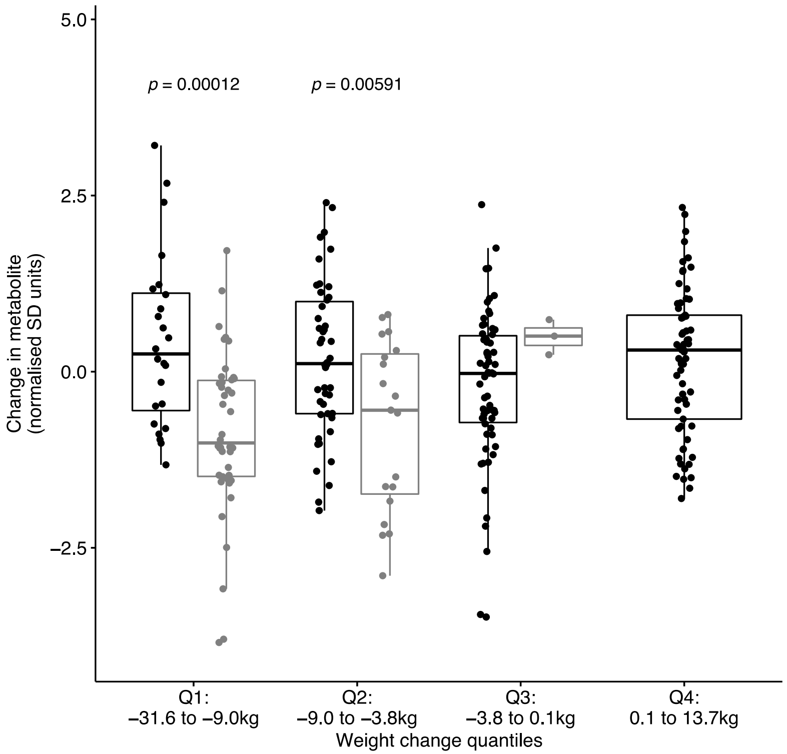

(b) Fructose

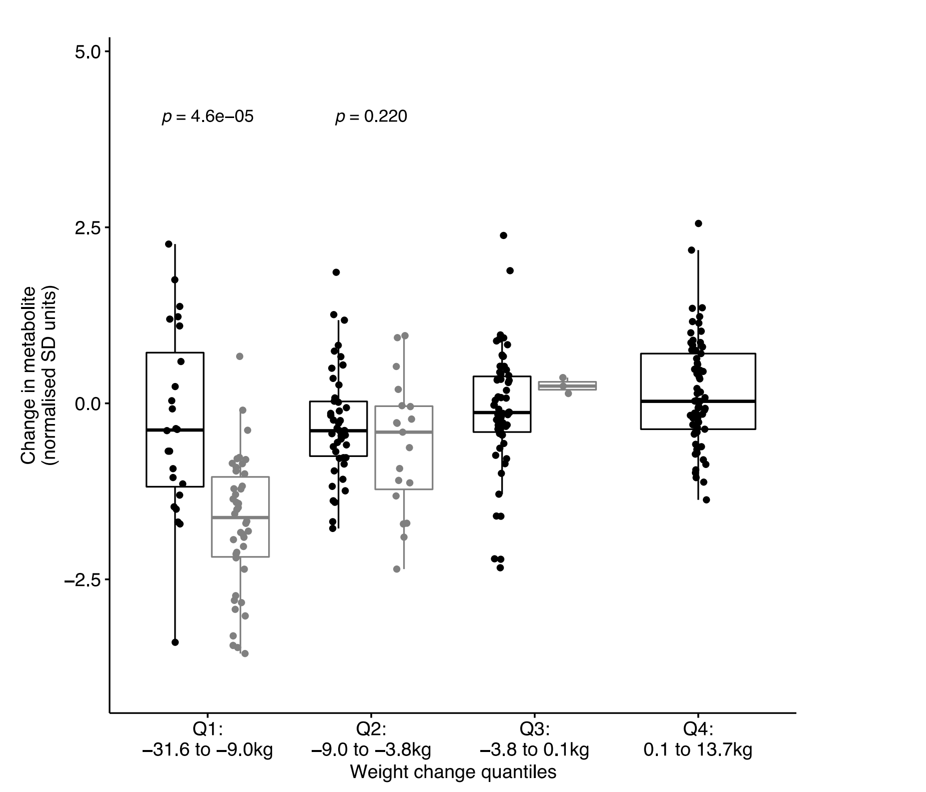

(c) Glucose

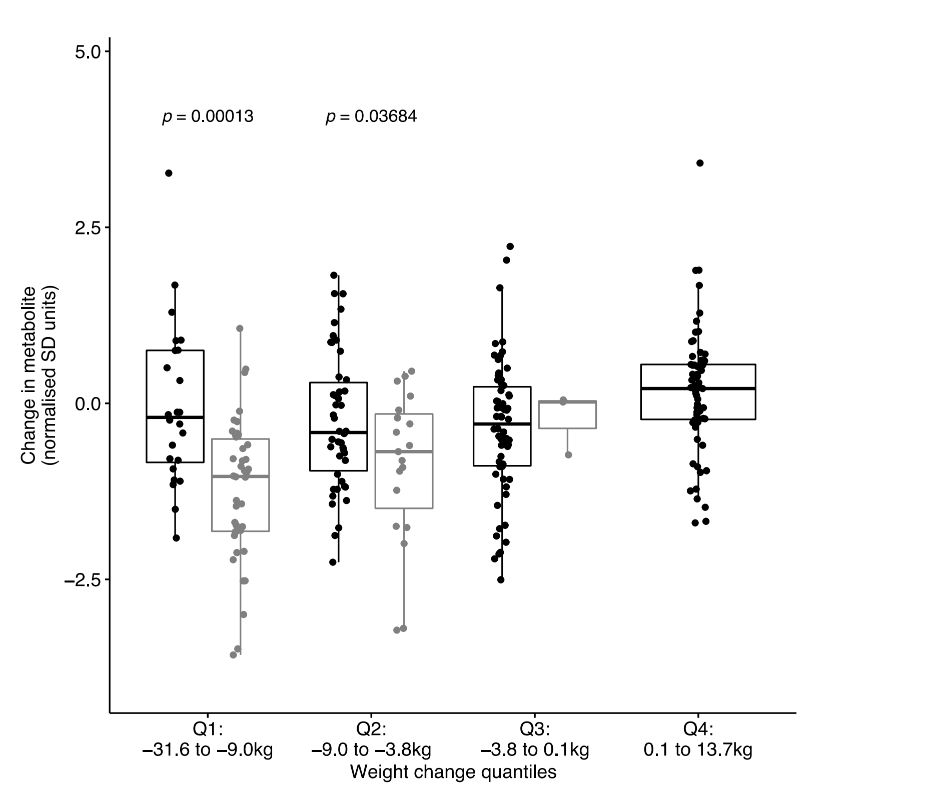

(d) Mannose

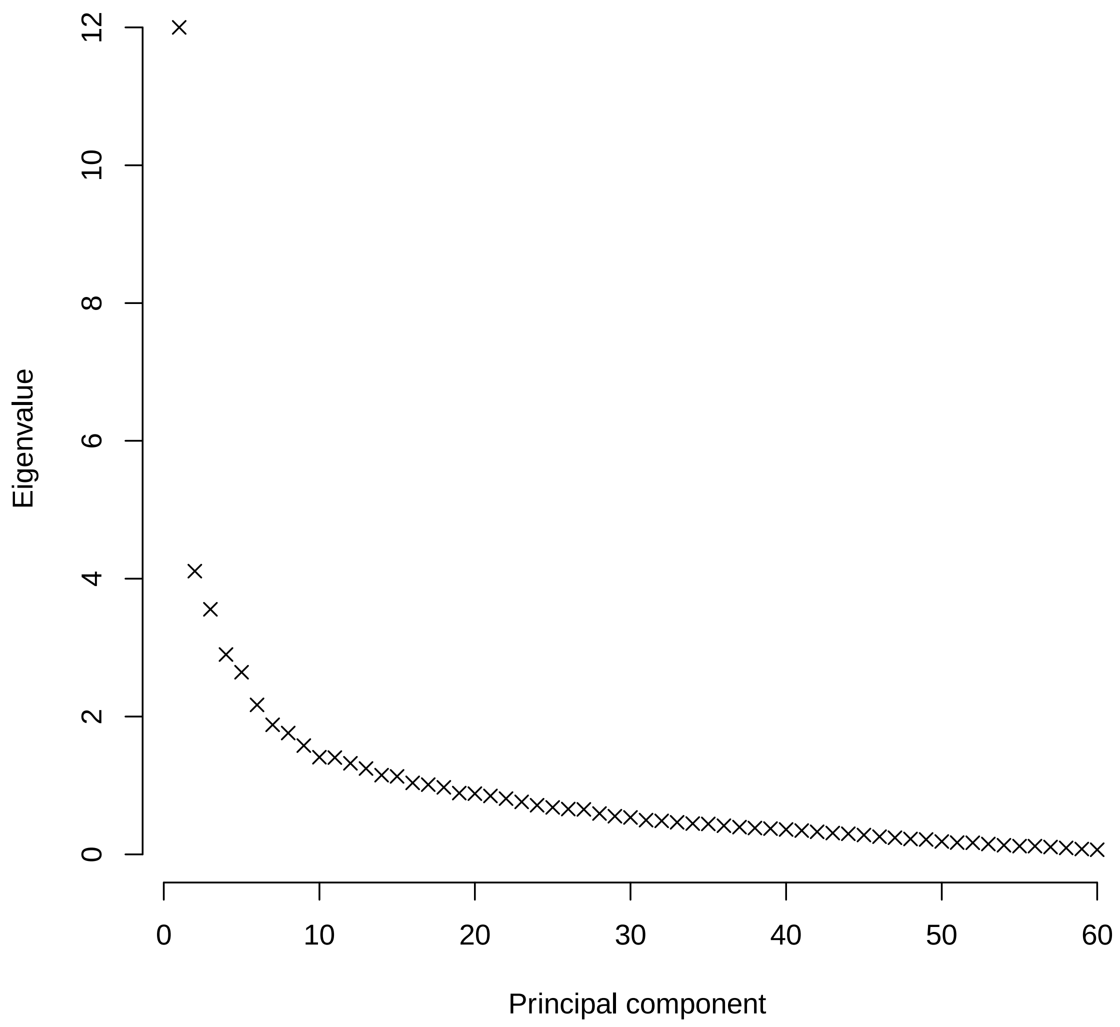

ESM Figure 5. Scree from PCA performed on data for the 61 associated representative metabolites

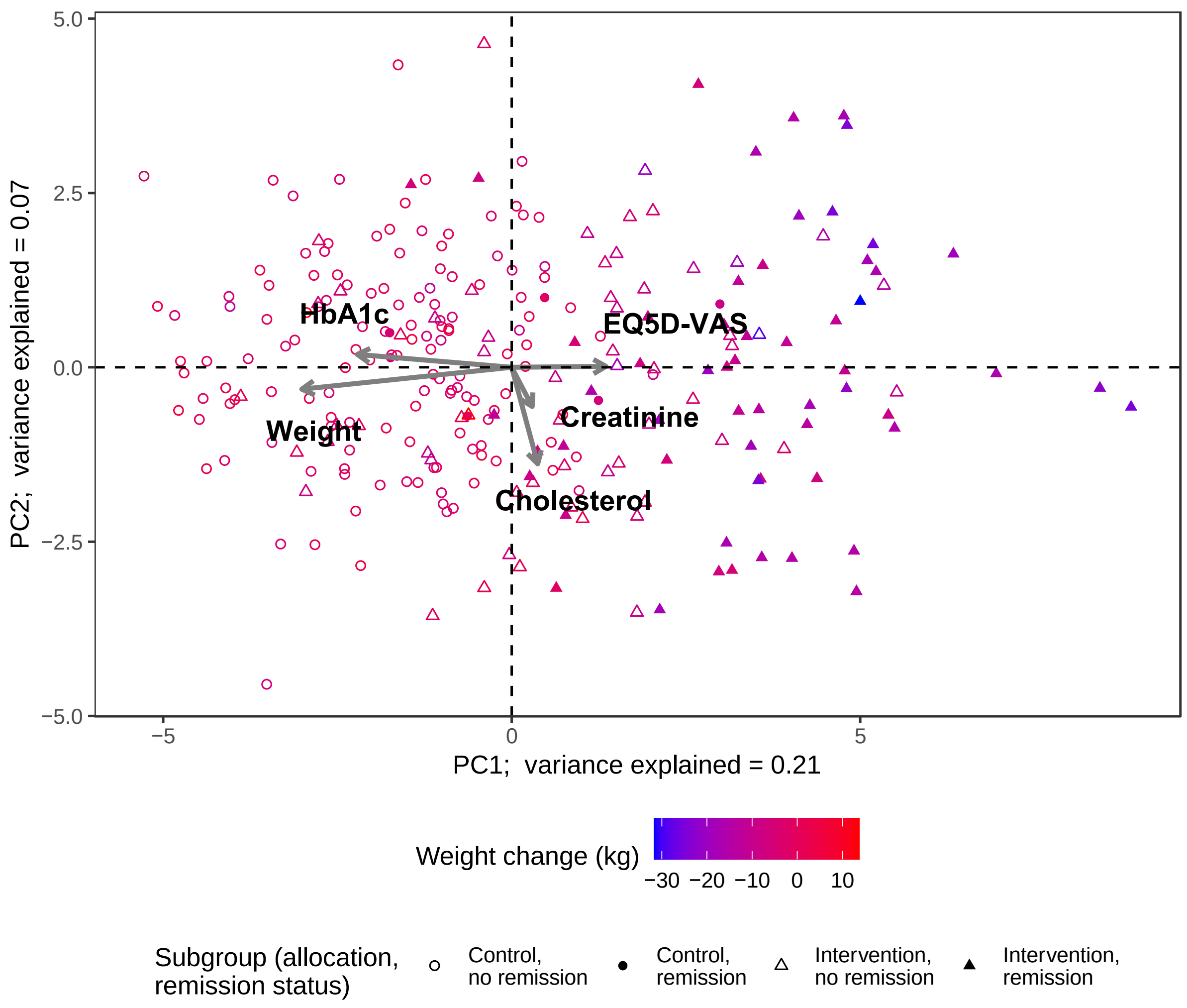

ESM Figure 6A. Principal component analysis (PCA) on samples using data for 61 associated representative metabolites after adjustment for covariates: biplot of general metabolic health.

Biplot to show relationship of PC1 and PC2 with change in a set of indicators of general metabolic health found to be correlated with PC1 and/or PC2 ($p<0.05$): Cholesterol = total cholesterol (mmol/l); Creatinine (umol/l); EQ5D-VAS = quality of life as assessed by EQ-5D visual analogue scale; HbA1c = glycated haemoglobin (mmol/mol). Further description of plot generation in **ESM Methods**. Change refers to the difference in measurements from the baseline timepoint to the 12-month timepoint. Weight change expressed in kilograms.

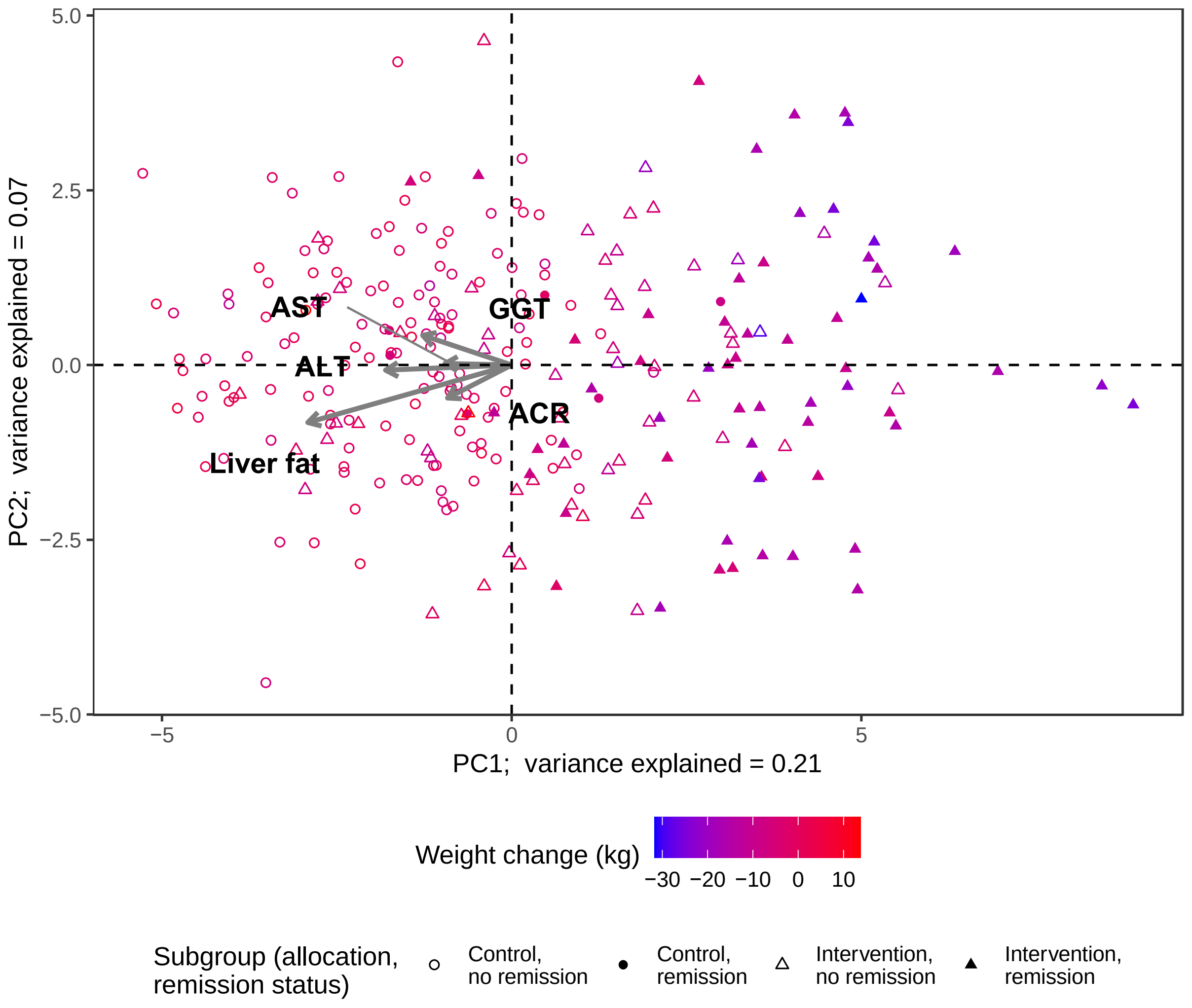

ESM Figure 6B. Principal component analysis (PCA) on samples using data for 61 associated representative metabolites after adjustment for covariates: biplot of NAFLD.

Biplot to show relationship of PC1 and PC2 with change in a set of parameters relevant for non-alcoholic fatty liver disease (NAFLD) found to be correlated with PC1 and/or PC2 ($p<0.05$): AST = aspartate aminotransferase (units/l), ALT = alanine aminotransferase (units/l), ACR = albumin-to-creatinine ratio (mg/mmol), GGT = gamma-glutamyl transpeptidase (units/L); Liver fat = liver fat percentage. Further description of plot generation in **ESM Methods**. Change refers to the difference in measurements from the baseline timepoint to the 12-month timepoint. Weight change expressed in kilograms.

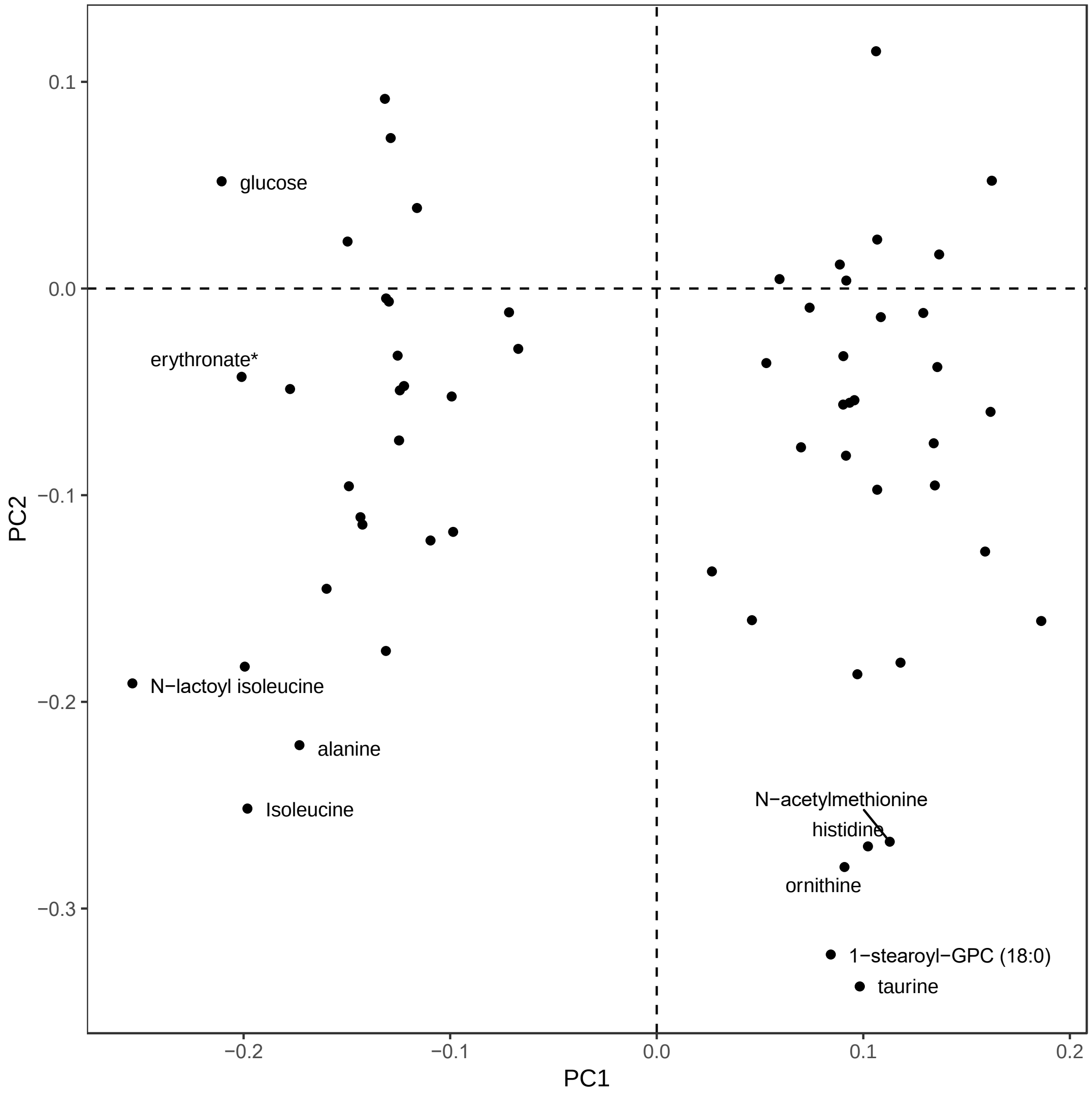

ESM Figure 7. Principal component analysis (PCA) on samples using data for 61 associated representative metabolites after adjustment for covariates: loadings plot.

PCA loadings plot to show the contribution of selected metabolites to the PCs. Labels added to identify variables with loadings > 0.2 or <-0.2 for one or both PCs.

* = indicates a compound that has not been confirmed based on a standard.

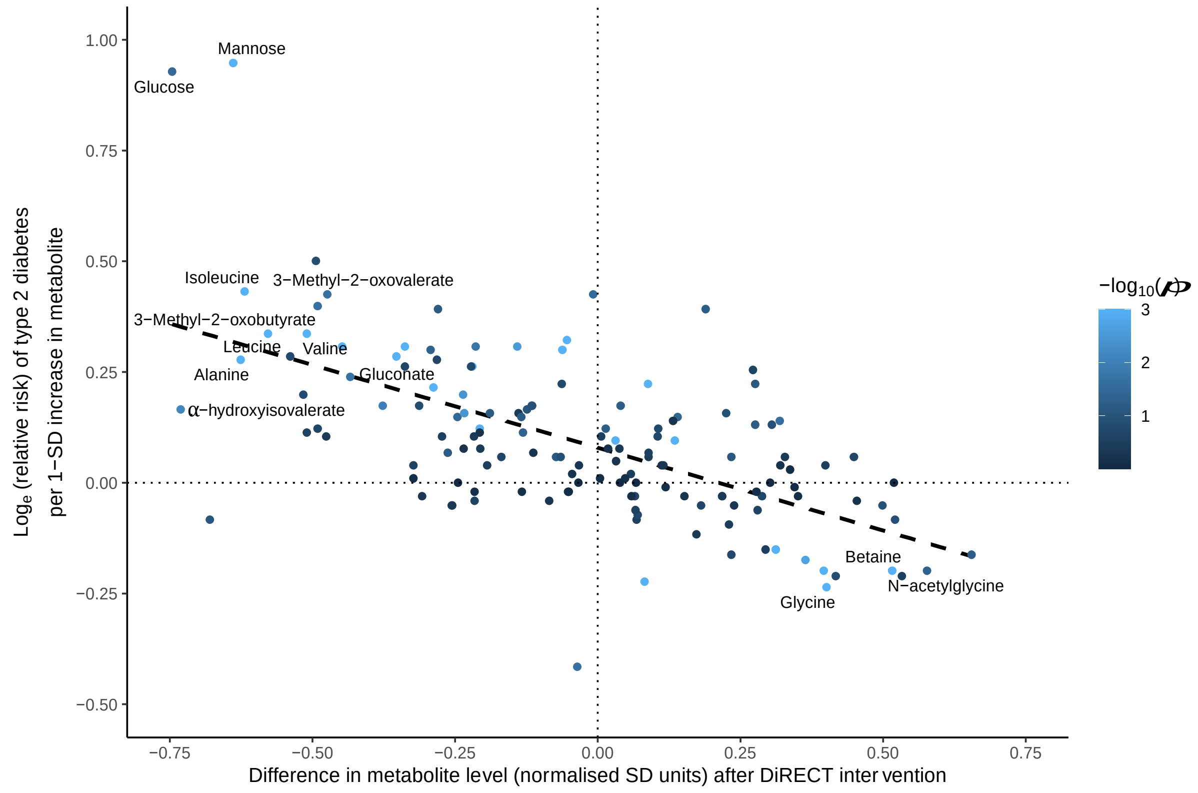

ESM Figure 8. Comparison of intervention and incident type 2 diabetes footprints including all matched metabolites.

Log$_e$ SRR estimates extracted from incident type 2 diabetes meta-analysis [18] plotted against mean intervention effects (βs) from our linear regression models (these βs represent the mean difference in metabolite levels at 12 months in the intervention group relative to the control group). Each point represents a metabolite that could be matched to meta-analysis results (143 matched in total); metabolites with Holm-corrected $p<0.05$ in the primary analysis are labelled. Points are coloured according to the SRR-associated *p* value in the meta-analysis. The correlation is −0.61 (95% CI −0.71, −0.50, $p=4.9\times10^{-16}$). **Figure 3** in the main text demonstrates the extent to which the changes we see in response to the intervention (with statistical support, $p<0.05$) are equal and opposite to those observed prior to development of type 2 diabetes. Here we include data for *all* metabolites that could be matched between our study and Morze *et al*. [18] to give an overall impression of the extent to which the DiRECT intervention delivers a general reversal of the metabolic profile of incident disease.

References

1. Soininen P, Kangas AJ, Würtz P, Suna T, Ala-Korpela M (2015) Quantitative Serum Nuclear Magnetic Resonance Metabolomics in Cardiovascular Epidemiology and Genetics. Circ Cardiovasc Genet 8(1):192–206. https://doi.org/10.1161/CIRCGENETICS.114.000216
2. Soininen P, Kangas AJ, Würtz P, et al (2009) High-throughput serum NMR metabonomics for cost-effective holistic studies on systemic metabolism. Analyst 134(9):1781. https://doi.org/10.1039/b910205a
3. DeHaven CD, Evans AM, Dai H, Lawton KA (2010) Organization of GC/MS and LC/MS metabolomics data into chemical libraries. J Cheminform 2(1):9. https://doi.org/10.1186/1758-2946-2-9
4. Evans AM, DeHaven CD, Barrett T, Mitchell M, Milgram E (2009) Integrated, Nontargeted Ultrahigh Performance Liquid Chromatography/Electrospray Ionization Tandem Mass Spectrometry Platform for the Identification and Relative Quantification of the Small-Molecule Complement of Biological Systems. Anal Chem 81(16):6656–6667. https://doi.org/10.1021/ac901536h
5. Hughes DA, Taylor K, McBride N, et al (2022) metaboprep: an R package for preanalysis data description and processing. Bioinformatics. https://doi.org/10.1093/BIOINFORMATICS/BTAC059
6. Stacklies W, Redestig H, Scholz M, Walther D, Selbig J (2007) pcaMethods - A bioconductor package providing PCA methods for incomplete data. Bioinformatics 23(9):1164–1167. https://doi.org/10.1093/bioinformatics/btm069
7. RStudio Team (2016) RStudio: Integrated Development Environment
8. R Core Team (2020) R: A Language and Environment for Statistical Computing
9. Brien C (2019) growthPheno: Plotting, Smoothing and Growth Trait Extraction for Longitudinal Data
10. Nagi D, Hambling C, Taylor R (2019) Remission of type 2 diabetes: a position statement from the Association of British Clinical Diabetologists (ABCD) and the Primary Care Diabetes Society (PCDS). British Journal of Diabetes 19(1):73–76. https://doi.org/10.15277/BJD.2019.221
11. Zhao S, Guo Y, Sheng Q, Shyr Y (2014) Heatmap3: an improved heatmap package with more powerful and convenient features. BMC Bioinformatics 15(Suppl 10):P16. https://doi.org/10.1186/1471-2105-15-S10-P16
12. Zhao S, Yin L, Guo Y, Sheng Q, Shyr Y (2020) heatmap3: An Improved Heatmap Package
13. Ward JH (1963) Hierarchical Grouping to Optimize an Objective Function. J Am Stat Assoc 58(301):236–244. https://doi.org/10.1080/01621459.1963.10500845
14. Murtagh F, Legendre P (2014) Ward's Hierarchical Agglomerative Clustering Method: Which Algorithms Implement Ward's Criterion? J Classif 31:274–295. https://doi.org/10.1007/s00357-014-9161-z
15. Taylor R, Al-Mrabeh A, Zhyzhneuskaya S, et al (2018) Remission of Human Type 2 Diabetes Requires Decrease in Liver and Pancreas Fat Content but Is Dependent upon Capacity for β Cell Recovery. Cell Metab 28(4):547-556.e3. https://doi.org/10.1016/J.CMET.2018.07.003

16. EL L, KG H, BS A, MJ C, JC M, R T (2011) Reversal of type 2 diabetes: normalisation of beta cell function in association with decreased pancreas and liver triacylglycerol. Diabetologia 54(10):2506–2514. https://doi.org/10.1007/S00125-011-2204-7
17. Taylor R (2008) Pathogenesis of type 2 diabetes: Tracing the reverse route from cure to cause. Diabetologia 51:1781–1789
18. Morze J, Wittenbecher C, Schwingshackl L, et al (2022) Metabolomics and Type 2 Diabetes Risk: An Updated Systematic Review and Meta-analysis of Prospective Cohort Studies. Diabetes Care 45(4):1013–1024. https://doi.org/10.2337/DC21-1705
